# *In vivo* tracking of grape marc biomarkers, bioconversion, metabolic tracers, and microbiota modulation in swine fed a polyphenol-rich extract diet

**DOI:** 10.1371/journal.pone.0325079

**Published:** 2025-06-10

**Authors:** Aly Castillo, Maria Celeiro, Beatriz Martínez-Vallespín, Laura Rubio, Diego Gonzalez-Iglesias, Rocío Facorro, Carmen Garcia-Jares, Jürgen Zentek, Marta Lores

**Affiliations:** 1 i-Grape Laboratory, Vía Isaac Peral 32, E-15890, Santiago de Compostela, Spain; 2 CRETUS, Department of Analytical Chemistry, Nutrition and Food Science, Universidade de Santiago de Compostela, E-15782, Santiago de Compostela, Spain; 3 Institute of Animal Nutrition, Freie Universität Berlin, Berlin, 14195, Germany; 4 Laboratory of Research and Development of Analytical Solutions (LIDSA), Department of Analytical Chemistry, Nutrition and Food Science, Universidade de Santiago de Compostela, E-15782, Santiago de Compostela, Spain; Yakin Dogu Universitesi, TÜRKIYE

## Abstract

This work evaluated the addition of the polyphenol-rich bioactive extract “e-Vitis”, derived from grape marc (the main by-product of the wine industry), into swine feed. This was performed with the aim of testing the *in vivo* bioavailability of functional compounds, mainly phenolics, through the digestive system and excreta, together with the detection of bioconversion products associated with gut microbiota improvements. Additionally, the palatability of e-Vitis feed was evaluated, as well as the absence of metabolites that could compromise its innocuity. Through a pilot trial, a global methodology for the extraction and direct analysis of polyphenols from samples of gastric contents, duodenum, jejunum, ileum, caecum, colon, faeces and urine of these animals was proposed for the first time. The extraction process of bioactive compounds from samples was carried out using the matrix solid-phase dispersion (MSPD) technique. High resolution QToF (quadrupole time-of-flight) mass spectrometry and metabolomics tools were employed to identify 112 biomarkers that clearly differentiated (*p* < 0.05) the two groups of pigs (with and without enriched feed). The results showed a bioamplifying effect of e-Vitis feed on bile acids in gastric contents, associated with reduced oxidative stress and enhanced liver protection. This was attributed to the capacity of grape marc polyphenols to encapsulate bile acids, facilitating their transport through the digestive system. Polyphenolic bioconversion pathways were also elucidated, detecting structures such as apigenin, davidigenin and isoliquiritigenin, metabolised from quercetins contained in e-Vitis feed. Likewise, several markers of gut microbiota metabolism, including hippuric acid, phenylacetic acid and phenylalanine, were identified in pigs fed e-Vitis, which were related to the intake of phenolic compounds. Therefore, this study provides a comprehensive methodology applied to various biological matrices (digestive system and excreta) to understand the metabolism of polyphenols and their value as bioindicators in the determination of effective doses of by-product addition in animal diets.

## Introduction

Better integration of livestock into the circular bioeconomy can be achieved by increasing the incorporation of agro-industrial by-products in the livestock feed ration. In contrast, correct but strict policies require higher feed standards, making the addition of by-products more complex. Regulations place their focus on quality feed, avoiding the addition of synthetic additives for growth enhancement, to improve animal development and health [1]. A variety of agricultural by-products such as corn gluten resulting from starch extraction, beet pulp derived from sugar production and defatted soya meal obtained as a result of oil processing are the main pillars of livestock feed [2]. This simple mix of ingredients primarily aims to boost the animal’s growth and fattening, making the addition of functional supplements necessary to enhance its nutritional profile [3]. Hence, one approach is to enrich feed with agro-industrial by-products naturally rich in polyphenols [4].

Phenolic compounds, a class of secondary metabolites present in plants, are produced to a great extent in response to both biotic and abiotic stresses [5]. These phytochemicals offer a comprehensive defence against the multifactorial nature of stressors by combining a variety of functional effects, making them an effectively superior tool to conventional unifactorial treatments [6]. This protective effect has sparked growing interest in their application, driving research into their antioxidant, antimicrobial, prebiotic, pharmacological, and other capabilities [7–9].

Recognised for their ability to counteract oxidative stress, which can cause serious alterations in cell metabolism, it has been reported that the ingestion of phenolic compounds by different animal species helps them to protect the intestinal mucosal barrier and improve nutrient absorption [10]. In addition, these compounds possess anti-inflammatory properties [11] that act through complex cellular pathways, contributing to the maintenance of a healthy gut environment. However, although polyphenol-rich foods show a positive effect on the gut microbiota, the precise mechanism of how they affect this microbiota remains unclear [12].

A particular characteristic of phenolics is that they exist in complex mixtures, making it difficult to analyse their pathway through the ingesting organism [13]. In addition, interactions between bioactive compounds and macronutrients, such as polysaccharides, fibres or proteins, may influence the bioaccessibility and bioavailability of phenolic compounds [14].When metabolised, a single phenolic compound can be transformed into multiple derivatives, in many cases completely different from the original compound, which can alter its biological effects [15]. This complexity has limited studies on the interaction of polyphenols with the gut microbiota in animal diets. On the other hand, it is known that these compounds not only provide antioxidant effects but also strengthen the immune system of animals and promote the growth of probiotics in species such as pigs, thus benefiting gut health [16]. Hence the interest in enriching farm animal diets with by-products rich in these compounds.

In the agricultural sector, grape marc is an important by-product whose valorisation has attracted the interest of farmers and stockbreeders, mainly for its potential reintroduction into the production chain [17–19]. Grape marc is composed of the remaining material from the winemaking process (skins, pips and stems), and meets two important requirements for use as an ingredient in animal feed. Firstly, this by-product is characterised by a high bioactive profile led by flavonoids and phenolic acids [20]. Secondly, this by-product represents a source of dietary fibres that may enhance digestibility and intestinal health of animals [12]. The marc represents 20–25% of the total grape used for wine, i.e., more than 10 million tonnes per year worldwide, and is a cause of concern for winemakers due to its complex management [21]. A common alternative to the reuse of grape marc is its implementation to improve soil quality due to its high macronutrient content. However, its direct and indiscriminate application in soils can result in a significant phytotoxic and antimicrobial effect due to the release of phenolic compounds, generating a negative impact on plant growth as undesired fermentation processes, not only wasting their intrinsic potential but also negatively modifying the environment where they are eliminated [22]. In response to this scenario, several studies have proposed ways to make the most of it, such as the use of marc extracts in farm animal feed [17,23,24]. One of the key challenges encountered in these studies is determining the optimal feeding doses for different animals while ensuring both their safety and the preservation of functional properties. This difficulty arises from the heterogeneous polyphenolic profile found in various types of marc and other agri-food waste [25].

Several approaches aim to standardise a methodology to quantify the effect of polyphenolic compounds in livestock. Techniques such as ultrasound-assisted extraction [26], liquid-liquid extraction [27] and accelerated solvent extraction [28] have been used for the extraction of thigh meat, caecal contents and organ tissue respectively, in the analysis of the effect of polyphenol intake in these animals. But animal specimens are very complex due to the heterogeneous nature of both solid samples, such as liver, colon or faeces, and liquid samples, mainly urine and plasma [29]. Most techniques require large amounts of solvents and/or reconcentration and purification steps, which negatively impacts the recovery of these analytes [30,31]. A simple process, applied to a wide range of solid, semi-solid and liquid samples, that combines the extraction and filtration stages in a single step is matrix solid-phase dispersion (MSPD). This technique, proposed by Barker *et al.* (1989), has been applied in numerous studies focused on the extraction of bioactive compounds, including phenolic chains, from various plants and organic matrices [32]. However, there is no data on the previous application of this technique to the recovery of polyphenolic compounds in biological samples of animal origin, such as those considered in this work.

Another promising development in understanding the impact of polyphenolic compounds on animal digestion is the ability to detect these compounds using advanced chromatographic and spectrometric technologies. Since the development of time-of-flight mass spectrometry two decades ago, it has been possible to detect and understand through metabolomic analysis the polyphenolic distribution in different plant and fruit extracts. Using algorithms based on its high detection capacity and mass accuracy, phenolic compounds have been identified in matrices where their potential to contain them was unknown [33]. Hence, there has been growing interest in applying this high-resolution technology to monitor the pathway through the digestive system of polyphenol intake in farm animals, their metabolism and potential effects.

This study assessed the impact of incorporating the bioactive white grape marc extract “e-Vitis” into swine feed by monitoring the bioavailability of its compounds, primarily phenolics, throughout the digestive system and excreta. Additionally, it examined the metabolic implications and biomarkers associated with gut microbiota modifications linked to animal welfare. For this purpose, the MSPD technique was applied for the first time to biological samples from the digestive system of pigs, providing a fast and simple sample treatment methodology with high selectivity towards the main bioactive compounds. In addition, a comprehensive analysis involving high-resolution chromatographic techniques coupled to quadrupole-time-of-flight (QToF) mass analysers was performed to provide a global view of the specific presence of the marker compounds and/or their metabolites in each segment of the digestive system.

## Materials and methods

### e-Vitis extract

The extract used in this study, named “e-Vitis” (*Vitis vinifera* marc extract) by the European NeoGiANT project from which it originates, was obtained from white grape marc of Albariño grape variety by i-Grape Laboratory Technology-Based Company (TBC) of the Universidade de Santiago de Compostela (USC). These grapes, characteristic of Rías Baixas appellation of origin, were grown and collected in the 2021 harvest, in O Salnés subzone, and processed in Mar de Frades winery. The aqueous e-Vitis extract was obtained using the patented medium-scale ambient temperature (MSAT) system [34], employing only GRAS (Generally Recognised as Safe) solvents at concentrations considered safe for consumption [35]. The resulting extract is a highly concentrated amber crystalline liquid, with a total polyphenol content of 15 grams per litre of extract (Data supplied by i-Grape). The individual profile of the main polyphenolic compounds detected is detailed in Table 1.

**Table 1:** Standard polyphenolic profile of e-Vitis^*^ extract.

Polyphenol	Concentration ± SD (mg_polyphenol_/L_extract_)
Gallic acid	19.0 ± 1.0
2,4,6-trihydroxybenzoic acid	2.2 ± 0.3
Caftaric acid	1.1 ± 0.3
Quercetin	2.8 ± 0.2
Quercetin-3-glucoside	40.0 ± 2.0
Quercetin-3-glucuronide	31.0 ± 2.0
Quercetin-3-rutinoside	3.6 ± 0.5
Kaempferol	0.8 ± 0.1
Catechin	123.0 ± 5.0
Epicatechin	120.0 ± 2.0
Epicatechingallate	45.0 ± 3.0
**∑** Procyanidines (B1 + B2 + C1)	259.0 ± 6.0

* Data supplied by i-Grape (raw data in S1 File)

### Feed nutritional profile

To feed the swine, a base mix of ingredients (Table 2) rich in protein, carbohydrates, and lipids, such as HP (High Protein Content) soybean meal and oil, corn, wheat, beet pulp and skimmed milk powder, was used. Other ingredients incorporated in the feed were the essential amino acids threonine, methionine, lysine, and tryptophan; lignocellulose as a source of fibre with a prebiotic effect; and calcium monophosphate and calcium carbonate salts as direct sources of calcium and phosphorus. The feed included an indigestible tracer (TiO_2_) used for determining the apparent nutrient digestibility which was one of the parameters of interest of the trial (not included in the current study). The basal feed was produced in a single batch of 40 kg, which was used throughout the entire feeding period. From this batch, 4.5 kg were separated and mixed with 300 ml of the e-Vitis extract (representing a 6.7% addition) using a horizontal rotary paddle homogenizer. The supplemented feed achieved a total polyphenol content of 1 g/kg, derived from the extract.

**Table 2:** Nutritional composition of pigs feed (as fed).

Ingredients	% (in mass)
Maize	29.99
Wheat	29.29
Soybean meal HP	11.40
Skimmed milk powder	10.00
Beet pulp	8.00
Soybean oil	4.50
Calcium monophosphate	0.83
Lysine	0.78
Calcium carbonate	1.07
Methionine	0.47
Threonine	0.54
Tryptophan	0.23
FU-Mineral	1.71
Lignocellulose	1.00
TiO_2_	0.20

The chemical analyses of the feed samples included the Weende components (crude protein, crude fiber, ether extract, ash, and nitrogen-free extract) and, in addition, starch, Ca and P. The analyses were in accordance with the methods published by the Association of German Agricultural Research and Analysis Institutes (VDLUFA). Thus, dry matter (VDLUFA III 3.1), crude protein (VDLUFA III 4.1.2 modified according to the determination of macro-N by means of a vario MAX CN analyser), crude fibre (VDLUFA III 6. 1.4), crude ash (VDLUFA III 8.1), ether extract (VDLUFA III 5.1.1), starch (VDLUFA III 7.2.1), Ca and P (VDLUFA VII 2.2.2.6) were analysed. In addition, the fibre fraction was analysed to determine amylase-treated neutral detergent fibre (aNDF), acid detergent fibre (ADF), and acid detergent lignin (ADL), using the Van Soest method as described by [36]. The method was adapted to use fibre filter bags instead of filter crucibles in an automated fibre analyser (ANKOM 2000 Automated Fiber Analyzer, USA). TiO2 was measured in the feed and ileal digesta according to the method described by [37].

### Animal samples

All animal experimental protocols were approved by the State Office of Health and Social Affairs Berlin (Landesamt für Gesundheit und Soziales Berlin, Germany, Reg. No. StN 014/22). In the view of animal welfare, all animals were slaughtered humanely by intracardiac pentobarbital injection, and all efforts were made to minimize suffering. The animal trial was performed at the Institute of Animal Nutrition, Freie Universität Berlin, Germany. All samples were obtained from a standardised trial with animals reared under identical and standard breeding conditions. Four male piglets with similar starting body weights (9.88 ± 0.06 kg) were randomly assigned at weaning day (42 ± 2 days of age) to two pens, each representing a separate group.

The reduced sample size, as part of a pilot trial, and its impact on the generalisability and statistical significance of the findings, should be viewed critically. This study undertakes to transparently discuss these limitations in our reports and to recommend that the results are interpreted as preliminary. Furthermore, the study has been designed with a rigorous methodology to ensure that the data, although preliminary, are robust and reproducible, laying a solid foundation for follow-up research. All piglets received the basal diet for 11 days and, after that, animals in one box received the polyphenolic extract enriched feed, while another box (control group) continued with the basal diet for three days. Feed was given *ad libitum* during the whole trial, regardless of diet, to all pigs included in the study. Daily monitoring of health status was carried out, paying special attention to the detection of digestive disorders. Feed consumption per piglet was estimated by taking the total amount of feed per pen for the period, subtracting the feed remaining and any losses, and then dividing the result by the number of piglets in each pen and the number of days in the period. Two weeks after weaning, the animals were slaughtered at an average body weight of 11.6 ± 0.59 kg for the group control and 12.0 ± 0.30 kg for the group receiving the e-Vitis extract, and samples of gastric content, jejunum, ileum, caecum, colon, faeces and urine were collected.

### MSPD extraction of biological samples

The main feed ingredients (beet and soya), whole feed and biological specimens were removed from storage at −20 °C, tempered and weighed in 1 g portions. The MSPD extraction process was based on the methodology of [38] with slight modifications as outlined below. In a mortar, 1 g sample was combined with 8 g of SiO_2_ (particle size 0.707 mm) and crushed for 5 minutes to a homogeneous paste. The extraction was carried out using a 15 mL TELOS® column containing a 10 µm x Ø16 mm PTFE filter, previously conditioned with a 1 g SiO_2_ layer. The sample was transferred to the column and gently compacted, adding a top layer of 1 g of SiO_2_ to act as an eluent distributor. Finally, the extract was eluted with 5 mL of a 50:50 methanol:water solvent mixture, maintaining a controlled flow rate of 1 mL/min by a regulating valve.

### Metabolites detection

The chromatographic method was based on [39] for the identification and quantification of bioactive compounds, with slight modifications as outlined below.. Samples were analysed by ultra-high performance liquid chromatography (UHPLC) coupled to a compact quadrupole time-of-flight (QToF) mass spectrometer (Bruker Daltonics). An Intensity Solo column (2.0 μm, 100 mm × 2.1 mm) maintained at a constant temperature of 40 °C was used. The mobile phase consisted of a solution of 4 mM formic acid in water (A) and methanol (B). Each acquisition was complemented by an injection of a standard calibrant for mass deviation correction containing NaOH at a concentration of 1·10^−3^ M in a 1:1 H_2_O:2-Propanol ratio and 0.2% formic acid. The total acquisition time was 20 minutes with a flow rate of 0.20 mL-min-1. The elution gradient started with a 95% (A)/5%(B) ratio for 0.4 min (calibrant injection interval), followed by a gradual increase of phase B in the following phase and time intervals (%:min): 30:4.5, 37:8.0, 50:9.0, 90:11.0, 90:14.0; reaching the initial conditions again at 16 min and hold for 4 min.

Using an electrospray ionisation (ESI) source and an AutoMS/MS mode acquisition with negative polarity, mainly pseudomolecular [M-H]^-^ ions were detected, according to the method described by [39] as outlined below. Briefly, an acquisition rate of 8 Hz spectra in 1s cycles through a voltage ramp of 10–105 eV and a filtered mass range of 20–1000 m/z was used. Compass HyStar and DataAnalysis version 5.1 (Build 201.2.4019) software were employed for data acquisition and preprocessing respectively. Undirected annotations were performed using the software MetaboScape 4.0.4 (Build 19). Using the SmartFormula tool, algorithms based on intensity, isotopic profile and mass error were applied for the identification of the multiple compounds acquired. A comprehensive calculation example is presented in Section 1 in S1 Appendix. For the generation of molecular formulae, the mass deviation error was set to 5 mDa, and mSigma value <50 in all cases. To confirm the identifications generated, they were checked against online databases of chemical compounds: National Center for Biotechnology Information (NCBI) [40] and Chemical Entities of Biological Interest (ChEBI) [41]. Analysis of the fragmentation products of the compounds was also carried out by *in silico* predictions using the Compound Crawler and MetFrag tools. Mass spectra were automatically compared with spectral libraries: MassBank of North America (MoNA) [42], Mass Bank European Data Base [43].

### Statistical analysis

Through MetaboScape® Version 4.0.4 (Build 19) using the T-REX 3D algorithm, the raw data (S1 File) of more than 200 chromatograms were filtered, categorising the different study groups. The MetaboScape® built-in statistical package was used to screen the results by means of an unsupervised principal component analysis (PCA) model, using Pareto as the scaling algorithm with a 10% cross validation mode and a minimum variance explanation of 98%.

For further analysis, all previously generated data were exported to the online software MetaboAnalyst 6.0 [44]. The analysis parameters are shown in the Section 2 in S1 Appendix. The data were represented through volcano plots with threshold *p* < 0.05. Permutation tests with 2000 replicates were performed to validate the accuracy of the multivariate statistical models and to rule out possible randomness (*p* < 0.05) in the generated separation. The set of potential biomarkers selected in the study group (swine fed by e-Vitis-enriched feed) fulfilled all criteria in both test and validation datasets. The global analysis of the different digestive stages, excreta samples, and enriched feed was carried out using Heatmapper [45] online software, distributing the biomarkers in a heat map and in dendrograms, taking Complete Linkage as the grouping method, and Manhattan distance as the method of measurement (raw data in S1 File).

## Results

A comprehensive non-targeted study was conducted to highlight the potential effect of the addition of e-Vitis extract on the feeding of swine and its transit through the digestive tract. The inclusion of the polyphenolic-rich extract in the feed showed an average intake of 0.49 kg/day for e-Vitis pigs compared to 0.46 kg/day for pigs fed the basal diet. However, due to the focus of the study on the metabolic effect generated by the intake of phenolic compounds in the diet of the swine, as well as providing a mechanism for extraction and global detection of markers of these effects, the data on body weight are not sufficient to show clear differences or to draw conclusions in this respect.

The present study focused on the identification of the metabolic effects generated by ingesting the enriched feed in comparison to swine fed a standard diet. The nutritional composition of the diet is shown in **Table 3**. The study was organised in several stages: first, multivariate analysis process to group and classify the different types of samples. Secondly, feed biomarkers were analysed in the upper digestive tract. Finally, in the lower digestive tract and excreta samples, polyphenolic biomarkers and their metabolites were followed up. The combined study of the results allowed to propose and discuss several metabolic pathways of interest and their connection with the potential benefits for piglets fed with white grape marc extract.

**Table 3:** Analysed nutrient content of pigs feed (as fed).

Content	(g/kg)
Dry matter	908.00
Crude ash	55.10
Ether extract	63.50
Crude protein	201.00
Crude fibre	48.50
Neutral detergent fibre	165.00
Acid detergent fibre	71.40
Acid detergent lignin	9.50
Starch	436.00
Calcium	8.00
Phosphorus	4.70

### Biomarkers selection: principal component analysis (PCA)

**Fig 1:**
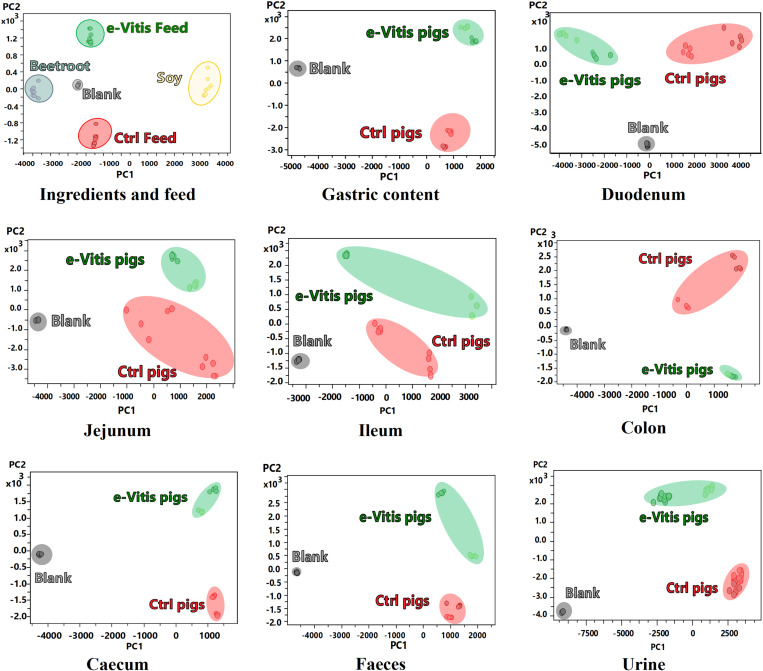
Principal Component Analysis (PCA) score plot of the feed, parts of the digestive transit, and excreta of pigs. Ctrl Feed: base feed; e-Vitis feed: feed enriched with e-Vitis; Blank: instrumental blank; e-Vitis pigs: pigs fed with enriched feed. Ctrl Pigs: pigs fed with base feed.

**Fig 2:**
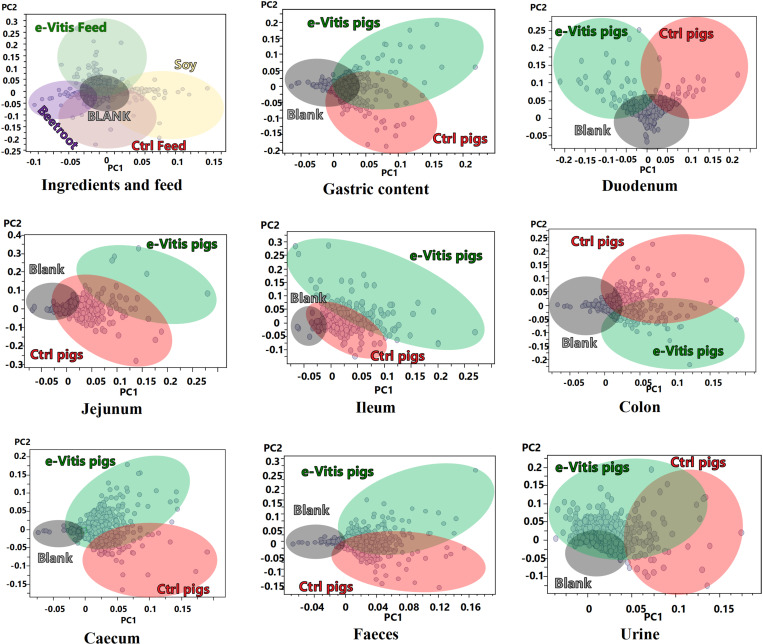
Principal Component Analysis (PCA) loadings plot of the feed, parts of the digestive transit, and excreta of pigs. Ctrl Feed: standard feed e-Vitis feed: feed enriched with e-Vitis; Blank: instrumental blank; e-Vitis pigs: pigs fed with enriched feed. Ctrl Pigs: pigs fed with standard feed.

Through the MSPD-UHPLC-QToF linkage, the chromatographic profile of each sample was generated, obtaining more than 4000 analytes distributed in the different study groups. The segmentation of these categories was performed by PCA summarised below by the scores (Fig 1) and loadings (Fig 2) plots. In this distribution, the main ingredients that constitute the feed, such as soybean and beetroot, as well as the base feed (Ctrl feed) and the enriched feed (e-Vitis feed), were represented. Similarly, different sections of the digestive tract of the swine (gastric contents, duodenum, jejunum, ileum, caecum and colon), as well as excreta (faeces and urine) were evaluated, divided between piglets that received the base feed (Ctrl pigs) and those that were fed the enriched feed (e-Vitis pigs). In each analysis, the response of the extraction solvent (methanol:water 50:50) was included as an instrumental blank.

Fig 1 presents a clear partitioning of the data, where each point reflects the chromatographic profile, and each colour corresponds to the same group of samples. This PCA process allows effective grouping of both experimental and instrumental replicates into small blocks. Fragmentation proved to be effective in most groups, with only a larger deviation observed between experimental replicates of jejunum and ileum samples. A more detailed explanation of the data segmentation, and the statistical and validation parameters used is summarised in Sections 3 and 4 in S1 Appendix.

In a second phase of analysis the markers of each group were segmented through loading plots (Fig 2). The distribution of these analytes in the area delimited by the principal components PC1 and PC2 correlates with the distribution of each sample in the PC1/PC2 analogue space of the score plot. For a better understanding of this interrelation, each area is highlighted with the colour of the corresponding characteristic group.

The selection criteria to determine the marker analytes of each group was those with PC1 and PC2 values presenting *p* < 0.05 obtained from the t-test and ANOVA analyses detailed in the Section 4 in S1 Appendix. Table 4 summarises the biomarker compounds identified in e-Vitis-enriched feed.

**Table 4:** Marker compounds identified in e-Vitis-enriched feed and their traceability in the digestive tract.

Nº	Name	Family	RT [min]	Formula	Mass [Da]	m/z	mSigma	Δm/z [mDa]	Fragments	e-Vitis	GC	D	J	I	CO	CE	F	U
1	Glucose + fructose	Carbohydrates	1.46	C₆H₁₂O₆	180.06	179.05	16.2	1.53	89-97-85-79-75	**X**	**X**	**–**	**–**	**–**	**–**	**–**	**–**	**–**
2	Mannitol + sorbitol		1.36	C₆H₁₅ClO₆	218.05	217.04	8.90	1.43	181-170-101-97-79	**X**	**–**	**–**	**–**	**X**	**–**	**–**	**–**	**–**
3	2-furoic acid	Carboxylic Acids	2.79	C₅H₄O₃	112.01	111.01	4.73	1.53	78-73-71-41-39	**X**	**X**	**–**	**–**	**–**	**–**	**–**	**–**	**–**
4	Gluconic acid isomer		1.37	C₆H₁₂O₇	196.06	195.05	7.16	1.33	129-87-85-75-59	**X**	**X**	**–**	**–**	**–**	**–**	**–**	**–**	**–**
5	Gluconic acid isomer		1.95	C₆H₁₂O₇	196.06	195.05	9.49	1.18	129-87-85-75-59	**X**	**X**	**–**	**–**	**–**	**X**	**–**	**X**	**–**
6	Gluconic acid derivate		1.37	C₁₈H₃₄O₁₈	538.17	537.17	33.35	0.20	179-159-129-75-59	**X**	**X**	**–**	**–**	**–**	**–**	**–**	**–**	**–**
7	2-isopropylmalic acid		6.08	C₇H₁₂O₅	176.07	175.06	8.16	1.15	157-115-113-103-85	**X**	**–**	**–**	**–**	**–**	**–**	**–**	**–**	**–**
8	Malic acid isomer		1.85	C₄H₆O₅	134.02	133.01	6.40	1.88	114-73-71-44-41	**X**	**X**	**–**	**–**	**–**	**–**	**–**	**–**	**–**
9	Malic acid isomer		1.43	C₄H₆O₅	134.02	133.01	6.41	2.04	78-74-72-71-68	**X**	**X**	**–**	**–**	**–**	**–**	**–**	**–**	**X**
10	Citric acid		2.81	C₆H₈O₇	192.03	191.02	13.72	1.40	129-112-111-85-75	**X**	**X**	**X**	**–**	**–**	**–**	**–**	**–**	**X**
11	Gallic acid glucoside	Flavonoids	4.31	C₁₃H₁₆O₁₀	332.08	331.07	14.85	2.03	334-283-269-170-125	**X**	**–**	**–**	**–**	**–**	**–**	**–**	**–**	**–**
12	Quercetin glucuronide		10.44	C₂₁H₁₈O₁₃	478.08	477.07	4.07	4.30	431-306-301-270-151	**X**	**X**	**X**	**–**	**–**	**–**	**–**	**–**	**–**
13	Catechin		5.6	C₁₅H₁₄O₆	290.08	289.07	2.40	0.48	289-286-245-203-132	**X**	**X**	**X**	**–**	**–**	**–**	**–**	**–**	**–**
14	Catechin glucoside acetate		5.6	C₂₄H₃₀O₁₁	494.17	493.16	21.43	9.07	289-245-221-205-203	**X**	**X**	**–**	**–**	**–**	**–**	**–**	**–**	**–**
15	Dihydroquercetin rhamnoside		9.79	C₂₁H₂₂O₁₁	450.12	449.11	28.56	1.05	449-447-303-285-285	**X**	**X**	**X**	**–**	**–**	**–**	**–**	**–**	**–**
16	Epicatechin		6.72	C₁₅H₁₄O₆	290.08	289.07	1.32	1.15	275-245-203-164-131	**X**	**X**	**X**	**–**	**–**	**–**	**–**	**–**	**–**
17	Kaempferol glucoside		11.17	C₂₁H₂₀O₁	448.01	447.09	10.56	0.84	285-256-255-228-227	**X**	**X**	**X**						
18	Quercetin galactoside		10.47	C₂₁H₂₀O₁₂	464.10	463.09	7.02	1.50	306-301-271-255-243	**X**	**X**	**X**	**–**	**–**	**–**	**–**	**–**	**–**
19	Quercetin glucoside		9.17	C₂₁H₂₀O₁₂	464.10	463.09	21.04	2.92	415-301-254-248-129	**X**	**X**	**X**	**–**	**–**	**–**	**–**	**–**	**–**
20	N-HODE dimer	Fatty Acids	13.48	(C₁₈H₃₂O₃)₂	592.47	591.46	10.62	1.07	295-293-277-171-113	**X**	**–**	**–**	**–**	**–**	**–**	**–**	**–**	**–**
21	N-HODE		13.48	C₁₈H₃₂O₃	296.23	295.23	16.02	0.10	283-229-211-183-44	**X**	**–**	**–**	**–**	**–**	**–**	**–**	**–**	**–**
22	N-HOTrE		13.28	C₁₈H₃₀O₃	294.22	293.21	14.55	0.65	276-275-121-113-96	**X**	**–**	**–**	**–**	**–**	**–**	**–**	**–**	**–**
23	N-TriHOME		12.57	C₁₈H₃₄O₅	330.24	329.23	11.87	0.06	328-209-171-36-34	**X**	**–**	**–**	**–**	**–**	**–**	**–**	**–**	**–**
24	Procyanidin B1	Procyanidins	5.42	C₃₀H₂₆O₁₂	578.14	577.14	12.87	2.11	451-425-289-161-125	**X**	**X**	**X**	**–**	**–**	**–**	**–**	**–**	**–**
25	Procyanidin B2		4.9	C₃₀H₂₆O₁₂	578.14	577.14	33.12	1.41	305-272-250-183-143	**X**	**X**	**X**	**–**	**–**	**–**	**–**	**–**	**–**

X: Detected, **–**: Not detected, e-Vitis: feed enriched with e-Vitis extract, GC – Gastric content, D – duodenum; J – Jejunum, I – Ileum, CO – Colon, CE – Caecum, F – Faeces, U – Urine.

### Classification of samples

**Fig 3:**
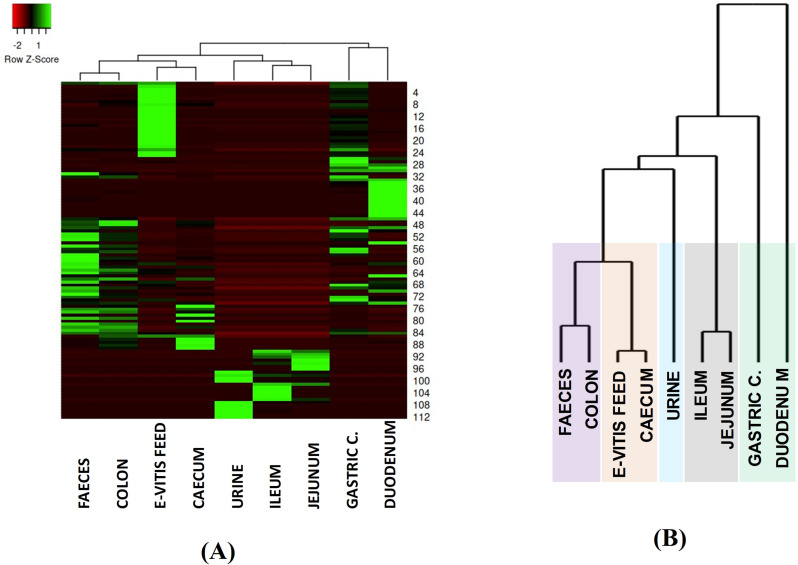
Comprehensive analysis of the transit of bioactive compounds and their metabolites generated by feed enriched with e-Vitis extract through the multiple regions of the digestive tract and excreta of pigs. Representation by: A) heat map and B) dendrogram classification.

To determine the effect of the enriched extract in contrast to the nutritional profile provided by the base feed, 112 compounds only contained in the PCA spaces characteristic of e-Vitis groups (with *p* < 0.05) and in the different stages of the digestive tract and excreta of the pigs under study were selected. Twenty-five of them correspond to the feed biomarkers shown in Table 4; the additional 87 markers appear in the samples of animal origin. As a graphical tool to visualise the pathway of these selected analytes, including the starting compounds of the e-Vitis extract and their metabolites and/or derived products, a heat map with dendrograms (Fig 3.A) is generated. This graphical tool facilitates the identification of clear clusters, as seen in Fig 3.B, highlighting obvious connections between adjacent stages, such as gastric contents and duodenum, jejunum and ileum, as well as colon and faeces. The urine shows a characteristic profile, with no direct connection to other segments, while the caecum shows a subtle correlation with the e-Vitis feed. In turn, as observed in the upper sectors of the heat map, this enriched feed exhibits a profile similar to the gastric content.

This analysis also enables the localisation of each of the 25 feed markers in the different digestive sectors and excreta samples, as also outlined in Table 4, making monitoring much easier. To obtain a comprehensive analysis of the metabolic processes involved, the identification of each family of analytes, their pathways and potential metabolites is presented, focusing on those that are most prevalent. As shown in Fig 3.A, a common trace can be maintained between the e-Vitis feed and the early stages of digestion, comprising gastric and duodenal contents, which in turn are closely related. However, in the subsequent digestive stages, there is not such an obvious correspondence, and instead new bands are seen in the heat map, generated by potential additional metabolic processes. Thus, a first analysis of the pathway of tracer compounds from the feed to the adjacent gastric and duodenal stages of the swine is established. To follow by an in-depth analysis of the metabolites in the lower digestive system and excreta samples.

## Biomarkers: feed and upper digestive system

### Feed

**Fig 4:**
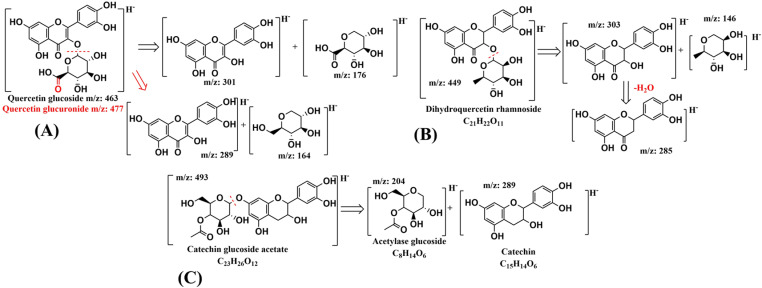
Fragmentation pathways of glycosylated phenolic compounds. A) Quercetin glucuronide and quercetin glucoside; B) dihydroquercetin-O-rhamnoside; C) Catechin glucoside acetate.

The identification of the 25 specific marker compounds of the enriched feed, classified by families of compounds, is shown in Table 4. Among these families, flavonoids and procyanidins predominate, headed by catechin, epicatechin and procyanidins B1-B2, polyphenols characteristic of grape marc from white grapes and already identified in e-Vitis (Table 1). Two quercetin derivatives were also identified, quercetin glucuronide (compound 12, 477 m/z) and quercetin glucoside (compound 19, 463 m/z), producing the typical 301 m/z ion due to the loss of the glucuronic group (−176 Da) and the hexoxide (−162 Da), respectively (Fig 4.A) [46]. Similarly, compound 15, dihydroquercetin-O-rhamnoside or astilbin (449 m/z) displays 303 and 285 m/z ions resulting from the loss of a rhamnose moiety (−146 Da) and subsequent loss of water (−18 Da) (Fig 4.B) [47].

Catechin and its glycoside linked to an acetate group (compounds 13 and 14, respectively) are identified by the catechin ion 289 m/z and its characteristic fragments 245 and 203 m/z resulting from the loss of the OH^-^ group in the C-ring, and subsequent rearrangement in the A-ring, as well as the breakage of the C-ring respectively. In the case of catechin glucoside acetate, fragmentation 493→289 due to the characteristic loss of glucoside from the −204 Da acetylase is evident (Fig 4.C) [48].

On the other hand, ions 181, 97, and 79 m/z, identified as glucose and the polyols sorbitol and mannitol respectively, have one of the highest distinct responses in the enriched feed, assumed by the addition of the extract. Grape marc is known to possess significant amounts of sugars such as glucose and fructose, as well as enzymes such as sorbitol dehydrogenase. It has been reported that post-harvest treatments of grapes, in which their water concentration is altered, can increase the conversion from glucose to fructose, generating sorbitol at an intermediate stage [49]. Furthermore, depending on the nature of the extractive solvent, enzymatic processes from glucose to fructose are favoured and the solubilisation of these sugars is reduced [50]. A modification of the enzymatic processes in the marc that increases the production of polyols and decreases the concentration of sugars can, therefore, be assumed.

Another notable group is that of the carboxylic acids composed of citric, malic and gluconic acids and their derivatives; common and highly present in grape marc [51]. Regarding fatty acids, long chain oxidation products of hydroxyoctadecadienoic (HODE) and trihydroxyoctadecenoic (TriHOME) acids are identified by the characteristic major ions 229, 183, and 171 m/z [52–54]. These oxylipin chains are associated with pest and pathogen protection processes in plants [55]. Different derivatives of HODE and TriHOME are also involved in anti-tumour processes, inhibiting metastatic processes [56], or as regulators of respiratory processes in diseases such as asthma [57]. Although these compounds have been identified in plants and in beverages such as beer, where they influence its characteristic bitter taste [53], their presence and function in grape marc is still emerging.

### Gastric content

The gastric content of e-Vitis pigs presented the closest match to the profile of the enriched feed in contrast to the other stages of the digestive tract, exhibiting an evident passage of the marker compounds. In a pseudo-target screening, using the spectral library generated from the 25 markers contained in the e-Vitis feed, 18 of these compounds were detected in the gastric contents (Table 4, GC column).

**Fig 5:**
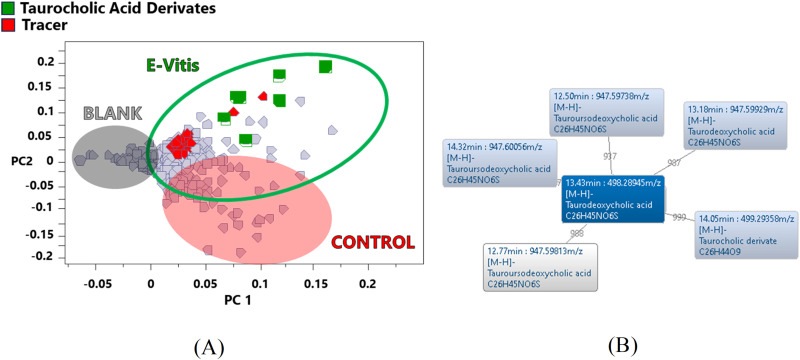
Distribution of tracers and taurine bile acid derivatives in the gastric contents of swine fed with e-Vitis. Display in the PCA plot (A) and their MS/MS correlations (B).

Except for gallic glycoside, all polyphenolic compounds were transported and detected with high intensity in the gastric fluid. Besides analysing the tracers, an untargeted search was also performed to identify the main indicators (*p* < 0.05) defining the PCA analysis group of the gastric contents of the study swine. Ion families 448, 446, 496, 498, 797 and 815 m/z (Table 5) were the most abundant in the PCA zone corresponding to the swine supplied with e-Vitis feed (Fig 5.A). Correspondingly, the MS/MS matches analysis (Fig 5.B) indicated a clear relationship between the mass profile of all these ions.

**Table 5:** Biomarker compounds identified in the gastric content of pigs fed with e-Vitis.

Nº	Name	Formula	RT [min]	Mass [Da]	m/z	mSigma	Δm/z [mDa]	Fragments	GC	D	J	I	CE	C	F	U
1	Taurodeoxycholic Acid	C₂₆H₄₅NO₆S	13.43	499.30	498.29	11.54	0.16	124-107-80	**X**	**X**	**–**	**X**	**–**	**–**	**–**	**–**
2	Taurohyodeoxycholic acid	C₂₆H₄₅NO₆S	14.86	499.30	498.29	11.93	0.37	124-107-80-44	**X**	**X**	**–**	**X**	**–**	**–**	**–**	**–**
4	7-oxotaurolithocholic acid	C₂₆H₄₃NO₆S	13.31	497.28	496.27	59.24	0.51	124-107-80	**X**	**X**	**X**	**X**	**–**	**–**	**–**	**–**
5	Glycoursodeoxycholic acid	C₂₆H₄₃NO₅	12.75	449.31	448.31	6.56	0.68	386-84-74	**X**	**X**	**X**	**X**	**–**	**–**	**–**	**X**
7	Glycocholic acid	C₂₆H₄₃NO₆	12.67	465.31	446.29	31.00	0.18	402-386-344-84-74	**X**	**X**	**X**	**X**	**–**	**–**	**–**	**X**
8	Cholic Acid Dimer	(C₂₆H₄₃NO₅)₂	13.02	816.57	815.57	5.92	1.18	425−407	**X**	**X**	**–**	**–**	**–**	**–**	**–**	**–**
9	Deoxicholic Acid derivate	–	13.13	798.56	797.57	–	–	425-407-389-80	**X**	**–**	**–**	**–**	**–**	**–**	**–**	**–**
10	Taurocholic Acid	C₂₆H₄₅NO₇S	13.32	515.29	514.28	7.61	0.49	124-107-80	**X**	**X**	**X**	**X**	**X**	**–**	**–**	**–**

X: Detected, **–**: Not detected, GC: gastric content, D: duodenum, J: jejunum, I: ileum, CE: caecum, C: colon, F: faeces, U: urine.

From this group of analytes, the most intense ions, 498 and 496 m/z, were designated by the SmartFormula tool as C_26_H_45_NO_6_S (498 m/z) and C_26_H_43_NO_6_S (496 m/z). Also, the mass spectra of each structure are contrasted by detecting the common ions 124, 107 and 80 m/z. Both structures and fragments are characteristic of taurine-conjugated bile acids, specifically taurodeoxycholic acid and isomers for the 498 m/z ion, and 7-oxotauroliotocholic acid for the 496 m/z ion. Reported fragments have been described as feature ions of taurine bile acids corresponding to 124 m/z (H₂NC₂H₄SO₃^-^), 107 m/z (CH₂CHSO₃^-^) and 80 m/z (SO₃^-^) [58]. Ion 514 m/z, with the presence of fragments 107 and 80 m/z, is clearly identified as taurocholic acid with formula C_26_H_45_NO_7_.

Fragments 797 and 815 m/z, sharing product ions 389,391, 405, 407 and 425 m/z, are related to the bile acid family. These ions are distinctive for dimers of cholic acid (815 m/z) and protonated deoxycholic acid (797 m/z) of the form [cholic+H- + cholic]- and [cholic+H- + deoxyholic]-, respectively [59]. Ions 448 and 446 m/z were labelled as C_26_H_43_NO_5_ and C_26_H_43_NO_6_. Both ions share a major ion, 74 m/z, common to compounds with glycine. In turn, these analytes show affinity through MS/MS matches with several bile acids, being particularly identified as bile acid glycosides, specifically glucodeoxycholic acid (448 m/z) and glycocholic acid (446 m/z).

### Duodenum

**Fig 6:**
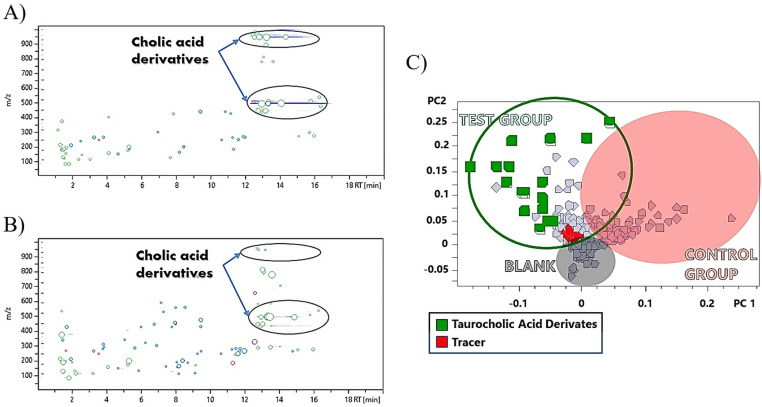
Distribution of tracers and taurine bile acid derivatives in the duodenum of swine fed e-Vitis feed. Topographical map (A) of the duodenal section and its correlation with the topographical map of the gastric contents (B), and Load diagram (C) of the duodenal section.

Duodenum analysis results in a strong correlation with the gastric content (Fig 3.B) and similarities with the enriched feed, as shown by the heat map (Fig 3.A). When tracing the 25 tracers (Table 4, D column), a reduction of the identified analytes compared to the gastric content is found, resulting in a total of 10 compounds. It is interesting to note that 9 of these analytes are phenolic structures, having a prevalence capacity in the digestive tract. Following a pseudo-targeted search, using the marker analytes in the gastric content as tracers, taurocholic acid derivatives were again detected in the duodenum. A comparison of the topographical map of the duodenum (Fig 6.A) with the respective gastric contents (Fig 6.B) indicates a higher abundance of taurocholic acid-derived compounds in the duodenum. Mainly in the zone of ions above 900 m/z where a higher density of analytes is exhibited. These compounds were used as markers to identify several derivatives of taurocholic acid by means of MS/MS matches (Table 6).

**Table 6:** Biomarker compounds identified in the duodenum of swine supplied with e-Vitis feed.

Nº	Name	Formula	RT [min]	Mass [Da]	m/z	mSigma	Δm/z [mDa]	Fragments	D	J	I	CE	C	F	U
1	Glycodeoxycholic acid isomer	–	13.14	449.31	448.31	–	–	386-330-84-74-72	**X**	**X**	**X**	**–**	**–**	**–**	**–**
2	Taurodeoxycholic Acid isomer	–	16.34	499.30	498.29	–	–	124-106-79	**X**	**–**	**X**	**X**	**–**	**–**	**X**
3	Taurodeoxycholic acid derivate	–	14.05	500.30	499.29	–	–	124-107-80	**X**	**–**	**–**	**–**	**–**	**–**	**–**
4	Taurocholic Acid derivate	–	15.77	516.31	515.30	–	–	124-96-80	**X**	**–**	**–**	**–**	**–**	**–**	**–**
5	Glycodeoxycholic acid dime	–	16.77	898.63	897.62	–	–	498-448-402-386-281-255-167-74	**X**	**X**	**X**	**–**	**–**	**–**	**–**
6	Glycodeoxycholic acid derivate	–	13.63	912.61	911.6	–	–	499-464-448-446-196	**X**	**X**	**X**	**–**	**–**	**–**	**X**
7	Tauroursodeoxycholic acid derivate	–	13.53	932.61	931.61	–	–	498-482-390-372-283-196-74	**X**	**–**	**–**	**–**	**–**	**–**	
8	Taurodeoxycholic acid derivate	–	12.50	948.61	947.60	–	–	500-498-496-124-79	**X**	**–**	**X**	**–**	**–**	**–**	**–**
9	Tauroursodeoxycholic acid derivate	–	12.44	964.60	963.59	–	–	514-498-484-464-124-106-74	**X**	**X**	**X**	**–**	**–**	**–**	**–**
10	Taurocholic derivate	–	12.41	980.59	979.59	–	–	514-498-482-446-122-113	**X**	**–**	**–**	**–**	**–**	**–**	
11	7-oxotaurolithocholic acid derivate	–	12.59	998.59	997.58	–	–	514-498-446-124-106-96-79	**X**	**–**	**X**	**–**	**–**	**–**	**–**
12	Apigenin 7-O-glucuronide	C_21_H_18_O_11_	10.81	446.08	445.07	0.998	29	269-113-74-175-59-85	**X**	**–**	**–**	**–**	**–**	**–**	**X**
13	Apigenin	C_15_H_10_O_5_	11.68	270.05	269.04	0.119	13.8	113-74-175-59-85	**X**	**–**	**–**	**–**	**–**	**–**	**X**

X: Detected, **–**: Not detected, D: duodenum, J: jejunum, I: ileum, CE: caecum, C: colon, F: faeces, U: urine.

The representation of these bile acids in the loading plot (Fig 6.C) appears as the main differentiating group in the area corresponding to the swine fed with e-Vitis. This supports the initial assumption of a wider availability of these compounds in other sectors of the digestive tract.

Medium-sized cholic acid derivatives between 400–600 Da were identified in the duodenum as isomers and derivatives of glucodeoxycholic and taurodeoxycholic acids. Compounds 1 and 2 (Table 6) show the fragmentations already seen in the gastric content (449→386→84→74 m/z y 499→ 124→107→80 m/z) for both acids. Compounds 3 and 4 present ions 499 and 515 m/z identified as derivatives of protonated taurodeoxycholic and taurocholic acids.

In the 800–1000 Da range, dimers and derivatives of taurocholic, glucodeoxycholic and taurodeoxycholic acids are detected, fragmenting into the monomeric ions 514, 448 and 498 m/z, together with the characteristic product ions 124 and 74 m/z.

Additionally, to the high response of bile acids, ions 445 m/z and 269 m/z stand out. Compound 13 (269 m/z) shows product ions 175 and 113 m/z, indicative of apigenin fragmentation [60,61]. The structure corresponding to the 445 m/z ion, on the other hand, shows a difference with apigenin of 176 Da, representative of the glucuronide group, while showing the same product ions. They are thus established as apigenin and apigenin glucuronide.

### Metabolites: Lower digestive system and Excreta

#### Jejunum.

Jejunum analysis provides less segmentation of the data than the upper sections of the digestive tract (Fig 1). Here, tracer compounds of the e-Vitis-enriched feed are no longer detected. This can be assumed due to the upstream absorption of the compounds, the degradation *per se* in the jejunum and the transport of nutrients into the blood through the villi of this section of the small intestine [62,63]. However, a clear clustering of the e-Vitis group can be discerned in the score plot (Fig 1), together with the presence of a small point cloud of characteristic analytes in the loading plot (Fig 2) that differentiates the groups studied (supported by the fit parameters depicted in S4.5 Section with R2Y>0.998, Q2 > 0.948, and *p* < 0.0001 [0/2000]). Table 7 details the differentiating compounds of the e-Vitis Group for the jejunum, highlighting a group of 7 compounds consisting of taurocholic acid and its derivatives, and bile acid glycosides.

**Table 7:** Biomarker compounds identified in the *jejunum* of swine supplied with e-Vitis feed.

Nº	Name	Family	RT [min]	Mass [Da]	m/z	Fragments	J	I	CE	C	F	U
1	Glycodesoxycholic acid derivative	Bile Acids	12.6	483.28	482.27	–	**X**	**X**	**X**	**–**	**–**	**–**
2	Glycodesoxycholic acid isomers		12.71	449.31	448.30	–	**X**	**–**	**–**	**–**	**–**	**–**
3	Glycodesoxycholic acid isomers		12.44	449.31	448.30	–	**X**	**–**	**–**	**–**	**–**	**–**
4	Tauronorursodeoxycholic acid		13.89	485.28	484.27	–	**X**	**–**	**–**	**–**	**–**	**–**
5	Isomers of taurodeoxycholic acid		12.92	499.29	498.28	–	**X**	**–**	**–**	**–**	**–**	**–**
6	Taurodeoxycholic acid isomer		14.93	499.29	498.28	–	**X**	**–**	**–**	**–**	**–**	**–**
7	Taurocholic acid isomer		14.27	515.29	514.28	–	**X**	**–**	**–**	**–**	**–**	**–**
8	Hippuric acid	Organic Acids	5.94	179.05	178.04	134-132-113-85-77-56-41	**X**	**X**	**–**	**–**	**–**	**X**
9	Indole-3-lactic acid		8.36	205.07	204.06	186-164-158-142-116	**X**	**X**	**–**	**–**	**–**	**–**
10	Isoliquiritigenin glucuronide	Phenolic glucosides	11.89	432.20	431.19	387-255-251-193-175-127-113-85-75-59	**X**	**X**	**–**	**–**	**–**	**X**
11	Conjugated glucuronide of davidigenin		12.11	434.21	433.20	387-257-193-175-127-113-85-75-59	**X**	**–**	**–**	**–**	**–**	**X**

X: Detected, **–**: Not detected, J: jejunum, I: ileum, CE: caecum, C: colon, F: faeces, U: urine

Another ion of interest, 433 m/z (compound 11 in Table 7), has fragments: 175, 113, 85 and 59 m/z, similar to those found in flavonoids like apigenin. This is a large flavonoid associated with a glycosylated derivative, showing a typical loss of −176 Da which generates the 257 m/z ion, consistent with a davidigenin glycoside. A known route for davidigenin formation involves dehydroxylation of flavanone glycosides via liquiritigenin or hydrogenation of flavanone glycosides via isoliquiritigenin [64]. This latter, isoliquiritigenin, is detected with the ion 431 m/z (compound 10 in Table 7), showing a product-characteristic ion profile in these glycosylated flavonoid chains already analysed.

**Fig 7:**
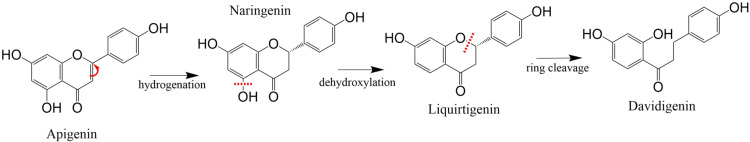
Metabolic pathway from apigenin to davidigenin.

Fig 7, based on [64], depicts a possible metabolic pathway starting from apigenin, generating naringenin by hydrogenation, followed by loss of a hydroxyl group by dehydroxylation to produce liquiritigenin, and finally, ring cleavage to form davidigenin.

An additional metabolite of note is represented by ion 204 m/z with product ions 186, 164, 158, 142, and 116 m/z; all previously reported as characteristic of the compound indole-3-lactic acid (ILA) [65]. This product of tryptophan metabolism has been linked to the intake of phenolic compounds from wine [66]. In turn, it shows high anti-inflammatory capacities, exhibiting an inhibitory effect on the release of inflammatory cytokines in intestinal epithelial cells [67].

One of the most remarkable compounds determined in the jejunum of the e-Vitis Group, as well as in ileum and urine, is represented by the 179 m/z ion. It has been identified as hippuric acid by comparison of its mass spectrum with the Human Metabolome Database (HMDB, identifier HMDB0000714), yielding its 7 product ions (134, 132, 113, 85, 77, 56 and 41 m/z). This organic acid is an acylglycine produced by the conjugation of benzoic acid and glycine, which is found as a normal component in urine [68].

### Ileum

Analogous to the jejunum, the ileum has a complex segmentation in its cluster distribution in the PCA plot (Fig 1). In contrast, a higher pooling is reflected in the load plot (Fig 2), along with higher R2Y>0.998 fit parameters and orthogonal distribution S4.4 Section), differentiating more clearly the group fed with enriched feed from the base feed. Bile acid derivatives are the most numerous biomarkers, as in jejunum (Table 8). They are shown with the 515 and 498 m/z ions derived from taurocholic acid, presenting the 124, 107 and 80 m/z fragments characteristic of these acids. The 498 m/z ion is specifically designated as a tauroursodeoxycholic acid (TUDCA) derivative, and exhibits important activities as an anti-inflammatory, neuroprotective and antioxidant agent [69]. Likewise, an oxotaurolithocholic acid derivative is shown, with the above-associated fragments 124, 96 and 79 m/z.

**Table 8:** Biomarker compounds identified in the ileum of swine supplied with e-Vitis feed.

N	Name	RT [min]	Mass [Da]	m/z	Fragments	I	CE	C	F	U
1	Glycohyocholic acid derivate	12.63	930.61	929.60	464−74	**X**	**–**	**–**	**–**	**X**
2	Taurocholic acid derivare	13.61	515.29	496.27	124-106-79	**X**	**–**	**–**	**–**	**–**
3	Taurocholic acid isomer	13.88	515.29	514.28	124-106-79	**X**	**–**	**–**	**–**	**X**
4	Taurodeoxycholic acid isomers	13.99	499.29	498.28	432-124-106-79	**X**	**–**	**–**	**–**	**–**
5	Hyodeoxycholic acid	14.57	438.30	437.29	409-392-373-355-345-160-96-79-44	**X**	**X**	**X**	**X**	
6	Oxotaurolithocholic acid derivate	15.85	489.16	488.15	453-162-124-96-79-44	**X**	**–**	**–**	**–**	**X**

X: Detected; **–**:Not detected; I: ileum, CE: caecum, C: colon, F: faeces, U: urine

In the ileum, larger bile acid chains are also evident, as in the duodenum (Table 6), with a new derivative of glycohyocholic acid with the ion 929 m/z and the product ions 464 and 74 m/z, already evident in these chains in the previous digestive stages.

By comparison with MassBank of North America (MoNA) databases, the identification of hyodeoxycholic acid (437 m/z) was obtained, validating the product ions 391, 373, 392, 355 m/z. This secondary bile acid, previously detected in bile salts from poultry and pigs [70], is associated with a decrease in LDL concentration, an improvement in hepatic cholesterol biosynthesis and modulation of cholesterol content in faeces [71].

#### Caecum.

PCA of the caecum (Fig 1 and Fig 2) indicates a clear differentiation between the groups studied, together with a defined concentration of analytes in the e-Vitis Group. This remarkable separation is reflected in the quality parameters of the OPLS-DA study (S4.6 Section) revealing the best set of parameters R2Y > 0.998, Q2 > 0.948, and *p* < 0.0001 (0/2000). In contrast, when compared to different segments of the digestive tract, the caecum exhibits a distinct singularity, characterised by a number of unique compounds that are not shared with the other samples analysed, observing only a small set of common analytes in the heat map (Fig 3). Thus, Table 9 details the distinctive compounds identified in the caecum of animals fed an e-Vitis-enriched diet, validated through the identification algorithm proposed in this study and confirmed in the different spectral databases. From the amino acid family, the identification of the compounds N-acetyl-L-methionine (190 m/z), ketoisocaproic acid (129) and their derivatives is proposed. Both compounds are common analytes derived from metabolic processes of *Escherichia coli* and their identification has been validated through the *E. coli* Metabolome Database (ECMDB). An additional compound identified, derived from the metabolism of bacteria that are part of the intestinal flora, is the auxin indolelactic acid (204 m/z). This organic compound is formed by the bonding of an indole ring with lactic acid, which is also identified with the ion 89 m/z.

**Table 9:** Biomarker compounds identified in the caecum of pigs fed e-Vitis feed.

Nº	Name	Family	RT [min]	Formula	Mass [Da]	m/z	Fragments	CE	C	F	U
1	Ketoisocaproic acid	Aminoacids and derivates	6.00	C₆H₁₀O₃	130.06	129.05	101-91-87-85-83-69-57-44	**X**	**X**	**X**	
2	Ketoisocaproic acid derivate		5.57	C₆H₁₀O₃	130.06	129.05	87-85-71-59-45-41	**X**	**X**	**X**	
3	N-acetyl-L-methionine		5.27	C₇H₁₃NO₃S	191.06	190.05	148-142-111-98-84-56-46	**X**	**X**	**X**	
4	Indolelactic acid	Auxins	8.41	C₁₁H₁₁NO₃	205.07	204.06	186-158-142-130-128-116-72-56-44	**X**			
5	Cholic acid derivate	Bile Acids	13.36	C₂₅H₄₂O₇	454.29	453.28	425-407-389-345-251	**X**	**X**		
6	Cholic acid derivate		14.46	–	454.29	453.28	425-407-389-283-61-44	**X**			
7	Cholic acid derivate		13.85	–	454.29	453.28	425-407-389-283-61-44	**X**			
8	Taurocholic acid isomer		13.61	C₂₆H₄₅NO₇S	515.29	514.28	514-407-390-372-124-106-95-79-61	**X**			
9	Cholic acid derivate		14.05	–	708.55	707.54	425-407-391-315-299-185	**X**			
10	Cholic acid derivate		13.57	–	784.58	783.57	425-407-391-315-299-185	**X**	**X**		
11	Cholic acid derivate		13.13	–	798.56	797.55	425-407-391-315-299-185	**X**			
12	Cholic acid derivate		13.56	–	800.58	799.57	425-407-391-315-299-185	**X**	**X**		
13	Cholic acid		12.78	C₂₄H₄₀O₅	812.54	811.53	407-387-293-171-11-80-59-44	**X**			
14	Bile acid derivate		13.18	C₄₈H₈₀O₁₀	816.57	815.56	425-407-361-321-59	**X**	**X**		
15	D-erythro-L-galacto-Nonulose	Carbohydrate derivative	1.54	C₉H₁₈O₉	270.09	269.08	179-119-113-101-92-89-78-71-59-43	**X**			
16	2-Hydroxybutyric acid	Carboxylic Acids	2.47	C₄H₈O₃	104.04	103.0401	59-57-45	**X**			
17	6-Hydroxyisocaproic acid		7.34	C₆H₁₂O₃	132.07	131.0714	131-129-113-89-86-85-12-71-69-44	**X**			
18	3-hydroxypentanoic acid	Fatty Acids	4.94	C₅H₁₀O₃	118.06	117.05	117-73-72-71-44	**X**			
19	Arachidonic acid		15.70	C₂₀H₃₂O₂	304.24	303.23	259-205-79-59	**X**			
20	Futalosine	Inosine derivative	3.50	C₁₉H₁₈N₄O₇	252.08	251.07	161-135-118-108-92-80-65-59	**X**	**X**	**X**	
21	Lactic Acid isomer	Organic Acids	1.56	C₃H₆O₃	90.03	89.02	–	**X**			
22	Lactic Acid isomer		1.79	C₃H₆O₃	90.03	89.02	–	**X**			
23	Lactic Acid isomer		2.77	C₃H₆O₃	90.03	89.02	–	**X**			
24	Maleylacetic acid		1.61	C₆H₆O₅	158.02	157.01	114-89-70-44	**X**			
25	Hydroxyphenylacetic acid	Phenolics	6.11	C₈H₈O₃	152.04	151.04	123-115-107-79-65-46	**X**		**X**	
26	Hydroxyphenyllactic acid		5.20	C₉H₁₀O₄	182.05	181.05	179-163-135-119-107-93-89-72-59-44	**X**			**X**
27	Daidzein		11.58	C₁₅H₁₀O₄	254.05	253.05	223-208-195-149-132-91	**X**			**X**

X: Detected, **–**:Not detected, CE: caecum, C: colon, F: faeces, U: urine

Indolelactic acid or indole-3-lactic acid displays intestinal probiotic functions such as antioxidant activity, immune regulation and reduction of inflammation [72]. Other derivatives of microbial metabolic processes are the analytes identified as 2-hydroxybutyric carboxylic acid (103 m/z) and futalosine (251 m/z). 2-Hydroxybutyric acid plays a role in regulating the gut microbiota and has a protective effect on liver damage [73]. In turn, futalosine plays a key role in the binding of menaquinone (vitamin K2) by bacteria in the intestinal tract [74]. Regarding the tracing of bile acids along the digestive tract, the caecum reveals a high number of cholic acid derivatives, including the acid itself (799 m/z) and validated with its characteristic fragmentation 425, 407, 391, 315, 299 and 185 m/z.

#### Colon and Faeces.

Table 10 illustrates the pooled analysis of the marker compounds present in colon and faeces from pigs supplied with e-Vitis-enriched feed. The analysis of these samples is performed together due to the close similarity of the analytes present in each one. Amino acids identified with ions 164 and 135 m/z presented the main differentiation both in the colon and faeces of e-Vitis pigs in contrast to those fed the base feed.

**Table 10:** Biomarker compounds identified in the colon of pigs fed e-Vitis feed.

Nº	Name	Family	RT [min]	Formula	Mass [Da]	m/z	mSigma	Δm/z [mDa]	Fragments	C	F	U
1	Phenylalanine	Aminoacids	4.05	C₉H₁₁NO₂	165.07	164.07	0.57	1.513	147-103-91-72	X	X	
2	Phenylacetic acid		9.46	C₈H₈O₂	136.05	135.04	4.35	1.93	91-85-59	–	X	
3	Cholic acid derivate	Bile Acids	12.8	–	454.30	453.29	–	–	425-407-389-361-345-59-44	X	X	X
4	Hyodeoxycholic acid derivate		12.91	–	784.58	783.58	–	–	409−391	X	X	
5	Cholic acid derivate		13.95	C₅₂H₈₀O₄	768.60	767.59	19.79	6.3916	407-391-375-299	X	X	
6	Hyodeoxycholic acid dimer		12.91	C₄₈H₈₀O₈	784.58	783.57	16.94	0.1538	409-391-377-110-59	X	X	
7	Cholic acid derivate		13.72	C₅₅H₈₈O₅	828.66	827.65	45.70	5.0089	433-407-391-265	X	X	
8	Taurine derivate		4.77	–	209.07	208.06	–	–	160-146-142-124-117-106-94-89-79-73-66-59-44	X	X	
9	Hyodeoxycholic acid derivate		14.16	–	438.29	437.29	–	.	409-391-44	X	X	
10	Cholic acid derivate		13.1	–	800.57	799.57	–	–	425-407-391-265	X	X	
11	Sugar chloride derivate	Carbohydrates	1.30	C₆H₁₃ClO₆	216.04	215.03	24.25	0.8582	181-154-145-128-104-96-92-89-78-59-34	X	X	
12	Mallic acid derivate	Carboxylic Acids	1.49	–	129.04	128.03	–	–	115-89-82-72-71-45	X	X	
13	Methylglutaric acid		5.01	C₆H₁₀O₄	146.05	145.04	6.02	1.7395	127-101-83-81	X	X	
14	Suberic acid		9.13	C₈H₁₄O₄	174.08	173.08	1.97	1.1414	129-11-109-83-57		X	
15	Sebacic acid		11.96	C₁₀H₁₈O₄	202.12	201.11	8.10	0.94	183-139-111-57	X	X	X
16	Pimelic acid		6.76	C₇H₁₂O₄	160.07	159.06	6.37	1.6758	141-115-97-95-81	X	X	

X: Detected, **–**:Not detected, C: colon, F: faeces, U: urine.

**Fig 8:**
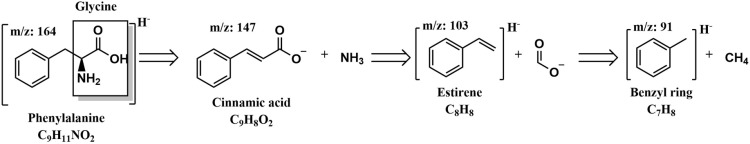
Phenylalanine fragmentation pathway.

The 164 m/z ion was characterised as phenylalanine due to its distinctive fragmentation 164→147m/z, resulting from the elimination of the N-terminal glycine as a neutral amide to form deprotonated cinnamic acid (Fig 8 based on [75,76]), which subsequently loses CO_2_ to form deprotonated styrene (103 m/z) [75]. Subsequently, its benzyl ring is observed (91 m/z), as well as its fragmentation into smaller structures (72 m/z) [76]. The ion 135 m/z, labelled as phenylacetic acid was associated by spectral matches with phenylalanine, as well as by querying the MoNA database.

The protective effect assumed by phenolic chains on taurocholic, and cholic bile acid derivatives is evident from gastric contents to this point, both in colon and faeces. A total of 8 bile acid derivatives are identified in colon and faeces through the common fragmentation 425→409→407→391 m/z that all these compounds present in general.

As mentioned above, carbohydrates such as glucose, fructose, mannitol and sorbitol stand out among the nutrients provided to the swine by the enriched feed. Thus, a chlorine adduct of these sugars (M + Cl)^-^ is detected both in colon and faeces with the ion 215 m/z and evidenced by the fragmentation 215→181 due to the loss of a Cl_2_ group (35 mDa), and leaving the ion 181 m/z corresponding to the compounds sorbitol and mannitol already recognised in the feed.

Another important family of metabolites identified are the carboxylic acid group, including suberic acid (173 m/z), sebacic acid (202 m/z), pimelic acid (160 m/z) and methylglutaric acid (145 m/z). These acids are associated with metabolic processes of fatty acid oxidation, through the activation of nuclear receptors that regulate energy metabolism. A modulating effect of phenolic chains such as catechin on these nuclear receptors has also been demonstrated, which may similarly lead to increased excretion of carboxylic acids [77].

#### Urine.

During the study of the various digestive stages in swine, a variety of compounds have also been identified as being present in urine. Starting with the profile of the enriched feed, malic and citric acids detected in e-Vitis feed are also found in urine. In the gastric content, bile acid derivatives are shared with urine; while taurocholic acid derivatives as well as the flavone apigenin and its glycosylated derivatives have been detected in duodenum and urine. The hydrochalcone davidigenin and hippuric acid associated with polyphenolic-containing diets were detected in both jejunum and urine. In the ileum and caecum, taurocholic compounds, amino acids and the isoflavone daidzein were observed and excreted in urinary samples. Finally, in the faeces and colon, cholic acid derivatives and a significant response of the carboxylic acid sebacic acid, also present in the urine, have been identified.

In contrast to the other digestive stages, urine like caecum shows an isolated profile represented in the dendrogram plot (Fig 3.B), however, it shows a stronger relationship mainly with the ileum and jejunum stages where a common zone is observed in the heat map. This similarity is assumed to be due to the fact that a large part of the metabolites present in urine are derived from the gut microbiota [78].

In terms of metabolic products, unique markers shown in Table 11 are present exclusively or with a significant (*p* < 0.05) higher response in the urine of swine supplied with e-Vitis feed. Besides the carboxylic acids previously identified in urine as citric, malic, hippuric, and sebacic acids; azelaic, succinic, α-ketoglutaric, itaconic and aconitic acids were also identified with ions 187, 117, 145, 129 and 174 m/z, respectively. Many of these organic acid derivatives are related to the consumption of diets with a high polyphenolic content. Especially, azelaic acid demonstrates significant levels in urine without undergoing metabolism at other stages of the digestive system due to metabolic modification induced by phenolic compounds. In previous studies in which hypercholesterolaemic pigs were fed with feed rich in phenolic compounds, higher levels of azelaic acid and sebacic derivatives were quantified in contrast to the control group [79].

**Table 11:** Biomarker compounds identified in the urine of pigs fed e-Vitis feed.

Nº	Name	Family	RT [min]	Formula	Mass [Da]	m/z	mSigma	Δm/z [mDa]	Fragments
1	Azelaic acid	Carboxylic Acids & Derivates	11.26	C₉H₁₆O₄	188.10	187.09	1.26	7.68	169-143-125-57
2	Succinic acid		1.8	C₄H₆O₄	118.03	117.02	5.27	2.14	99-74-73-55
3	Ketoglutaric acid		1.51	C₅H₆O₅	146.02	145.01	7.22	1.66	129-101-73-57
4	Itaconic acid		1.74	C₅H₆O₄	130.03	129.02	5.76	1.82	126-115-88-85-74-71-59
5	Aconitic acid		1.76	C₆H₆O₆	174.02	173.01	9.89	1.77	157-129-11-87-85
6	Benzoic acid	Phenolics compounds	8.66	C₇H₆O₂	122.04	121.03	4.49	1.33	119-101-79-77-71

## Discussion

Among the key factors addressed about the addition of functional by-products in feed was palatability, as animals could develop neophobic behaviours (aversion to a new ingredient). While the animals were fed e-Vitis feed “*ad libitum*”, the intake to satiety denotes that there was no negative impact on oral perception during the feeding trial. Likewise, one of the main complexities in the addition of functional compounds in farm animal diets is the determination of parameters indicating their effective action on animal welfare. Monitoring the bioavailability of these bioactive components along the digestive tract, such as their mechanisms of microbiota modification and bioconversion processes, can provide information on the actual functional action. Through in-depth monitoring of the transit of mainly phenolic compounds in the digestive tract and excreta of swine, potential mechanisms of action of these powerful antioxidants were revealed.

Starting in the upper digestive stages of the e-Vitis pigs, a high prevalence of phenolic compounds ingested from the enriched feed was detected. In the gastric content, 10 phenolic compounds were identified, led by catechin, epicatechin and procyanidins as glycosylated and glucuronidated derivatives of most of these compounds as well as kaempferol and quercetin. In the case of the compounds catechin, epicatechin, procyanidins and derivatives, together with the wide range of phenolic compounds contained in grape marc, several functional activities have been shown in the digestive system and intestinal microbiota [80]. Moreover, in line with the multi-objective approach pursued with the administration of the enriched feed, these three compounds show a significant synergistic action in different functional interactions such as the inhibition of food contaminants and potential coadjutants in therapies for the treatment of cancers [81,82]. Likewise, kaempferol and quercetin glycosides and glucuronides have been proposed to be more effective, in terms of bioavailability and absorption, than their free forms, being associated as inhibitors of low-density lipoprotein oxidation by scavenging reactive oxygen species (ROS) and anti-inflammatory effects in humans, although their mechanism of action *in vivo* analyses is limited [83].

In relation to the metabolic effects detected in the gastric contents of e-Vitis pigs, an increase in the concentration of cholic acids, including their glycols and taurine conjugates, is identified. Although the hypothesis of a common and limited reflux into the stomach supported by some studies [84–86] may explain this finding, reflux as a consequence of anaesthesia [87] or as a post-mortem phenomenon cannot be dismissed. On the other hand, the consumption of polyphenolic compounds has been linked to an increase in bile acid levels without causing harmful effects [88]. This amplifying effect is attributed to the sequestering action of polyphenols on bile acids, especially in polyphenolic compounds present in grapes, such as catechin and epicatechin [89]. Although the exact mechanism remains uncertain, it is suggested that this binding is due to a regio-specific interaction between polyphenols and bile acids [90]. Polyphenols would thus form a hydrophobic space that traps bile acids. A similar approach suggests that the binding of polyphenols to bile acids occurs through hydrogen bonds and hydrophobic interactions, reducing their solubility and, therefore, their bioavailability [91]. This encapsulation process could explain the higher concentration of bile acids in the gastric content of e-Vitis-fed pigs. By limiting the ability of the proximal small intestine to reabsorb them or by preventing duodenal bacteria from metabolising them, triggering their accumulation [92,93]. As a result, bile acids are more widely distributed throughout the digestive system, improving their bioavailability for bacterial deconjugation at later stages [94]. The higher distribution of bile acids is associated with a reduction in plasma cholesterol [89], an increased proliferation of bacteria capable of deconjugating glycine [95], as well as a prebiotic effect [96]*.* These results are consistent with the findings presented here, where the bile acid family is the most prevalent in the later stages of the digestive tract of pigs fed e-Vitis.

The detection of phenolic structures like apigenin and its glucuronide derivatives in the duodenum of the swine fed the enriched feed is also remarkable. This flavonoid stands out for its high antioxidant and anti-inflammatory potential [97]. Both apigenin and its glucuronides are related to the inhibition of inflammatory processes induced by lipopolysaccharide, as well as promoting cholesterol flux from cells and decreasing intracellular triglyceride content [98,99]. The origin of these polyphenolic and glycosylated compounds, which are not present in the e-Vitis feed, can be deduced as a bioconversion of polyphenols ingested. Significant modifications of these phenolic structures have been reported, mainly in flavonoids, in digestive processes, generating new flavonoid compounds [100]. Glycosylated flavonoid such as quercetin derivatives are affected by gastric pH, degrading and becoming more stable as glycosylated apigenin and kaempferol [101].

This behaviour is further supported when new bioconversion products like davidigenin glycoside and isoliquiritigenin glucuronide derived from previously restructured phenolic compounds are detected downstream of the duodenum, including jejunum, ileum and urine. Dehydroxylation and hydrolysis processes of polyphenols have been reported from the microbiota of the human digestive tract [102]. Therefore, it is feasible to assume that bioconversion of longer compounds, such as apigenin and quercetin glycosides, detected in the duodenum, originate from these compounds. Similar bioconversions have been documented in *in vivo* assays in rats [64] and in human digestive models [101]. Both davidigenin and isoliquiritigenin have demonstrated significant anti-inflammatory and antispasmodic action, inhibiting abnormal contractions in the duodenum and ileum [103].

Minor phenolic structures like hydroxyphenylacetic acid, daidazein and hydroxyphenyllactic acid are detected in the caecum, faeces and urine of e-Vitis swine. These compounds and related acids have been found to be strongly linked to the consumption of phenolic compounds, generating mediations in the bacterial metabolism of the digestive system. In particular, the isoflavone daidzein is of special nutraceutical interest due to its outstanding antioxidant activity. Daidzein has shown inhibitory effects on lipid peroxidation and oxidative stress, thus contributing to improve the welfare of the animals to which it is fed. Furthermore, this predominant detection in the excretory stages has been reported previously, noting that most of this isoflavone is eliminated in the faeces, while up to 30% of the daidzein consumed can be recovered in the urine [104]. Urinary excretion of phenolic acids, such as 3,4-dihydroxyphenylacetic acid and p-hydroxyphenyllactic acid, has been associated with the consumption of diets rich in polyphenolic compounds resistant to depolymerisation in the stomach, presenting a higher bioavailability in posterior stage of the digestive tract where they are absorbed as smaller phenolic acids [105,106].

According to these smaller phenolic structures, the detection of the amino acids phenylacetic acid and phenylalanine in faeces and colon of e-Vitis pigs is detailed. This family of phenylacetic auxin-derived acids has been established as markers in rutin- and quercetin-rich diets, being the main metabolites of microbial degradation of these phenolic compounds. In *in vivo* assays, their presence has been demonstrated in the colon and urine of rats fed high doses of quercetin and rutin, quantifying their antioxidant functionality mainly due to induced microbial metabolism rather than the direct effect of the polyphenols originating from this metabolism [107].

One of the compounds established as an indicator of the intake of diets with high polyphenolic content is hippuric acid. This metabolite, present in both the ileum and urine of e-Vitis swine, is considered a marker for the catabolism of dietary polyphenols. It is associated with the ingestion of foods or beverages with a high polyphenol load, showing elevated concentrations of this acid in both serum and urine [108–110]. Although its identification is mainly associated with urinary fluids, rats with a high intake of quercetin-4’-O-glucoside showed high concentrations of hippuric acid and derivatives in parts of the digestive tract such as jejunum and ileum, and also in urine and plasma [111]. Hippuric acid demonstrates several bioactivities such as antibacterial, fungicidal and cytotoxic; it maintains the acidity of urine and is used in pharmaceuticals for the treatment of urinary tract disorders against *E. coli*, enterococci and staphylococci pathogens [112].

In this final stage of metabolite monitoring throughout the digestive system and excreta, the absence of compounds that could generate an adverse action in e-Vitis pigs is relevant to note. Therefore, among the 112 analytes identified, there were no indications of compounds or metabolites that could compromise the safety of e-Vitis feed. Likewise, it is important to note that the constraints arising from the limited number of animals in the pilot trial were addressed through the analysis of a large size of samples from the digestive system and excreta, complemented by rigorous metabolomic analysis and extensive bibliographic support. This allowed metabolism and fragmentation pathways to be proposed that provide a comprehensive understanding of the effect of this functional feed in pigs. In addition, this pilot study is expected to drive the exploration of the mechanisms of interaction of phenolic compounds on animal welfare, the analysis of optimal addition levels in a larger number of animals, and the development of multi-target treatments for pathologies resistant to conventional single-target treatments.

## Conclusions

The functional effect of adding e-Vitis extract, derived from white grape marc and rich in polyphenolic compounds, to swine feed was evaluated. The MSPD extraction technique proved to be highly efficient in recovering bioactive compounds from various feed ingredients and biological samples (digestive system and excreta) from swine. The metabolomic study using UHPLC-QToF identified 25 feed tracer compounds and 87 metabolic markers differentiating between swine fed the standard diet and those fed e-Vitis-enriched feed. The predominance of more than 80% of flavonoids and procyanidins from e-Vitis, in the gastric and duodenal digestive system, revealed a bioamplifying effect on bile acids. This action on acids such as tauroursodeoxycholic acid (TUDCA) during the early stages of digestion increased its bioavailability in later phases of the digestive system, with implications for microbiota modulation. Also, the detection of markers of microbial metabolism induced by the ingestion of phenolic compounds, such as phenylacetic acid, phenylalanine, daidzein, hydroxyphenyl-lactic acid and hippuric acid, indicated a potential prebiotic effect in swine fed e-Vitis.

As part of a pilot study with preliminary results, this research seeks to lay the groundwork for future studies with larger numbers of animals and longer feeding periods to consolidate these findings. Nevertheless, this research represents the first comprehensive metabolomics approach to the study of polyphenol-enriched feeds in farm animals, proposing a unified method to extract and analyse markers across plant-derived ingredients, feed, digestive systems and excreta. The results provide valuable insights into the mechanisms of phenolic compounds in animal health and identify potential biomarkers to measure the efficacy of feed doses.

## Supporting information

S1 File Raw quantification data, graphs and statistics. (XLSX)

S1 Appendix Metabolomic identification, statistical analysis and study validation. (DOCX)
