# Supplementary material for: In vivo tracking of grape marc biomarkers, bioconversion, metabolic tracers, and microbiota modulation in swine fed a polyphenol-rich extract diet
Source: PLoS One. 2025 Jun 10;20(6):e0325079. doi: 10.1371/journal.pone.0325079 (PMC12151431; doi:10.1371/journal.pone.0325079)
Supplement: S1 Appendix — (DOCX) [file pone.0325079.s002.docx]

**S1 Appendix**

**Metabolomic identification, statistical analysis and study validation**

**Section 1. Metabolomics identification: Beyond tentative approaches**

After isolating the marker analytes of each group, they are identified by a first metabolomics approach. Through the use of analytical databases and spectral libraries, 112 compounds are tentatively named to be refined for a complete characterisation. This screening is performed by means of a rigorous calculation algorithm, taking as main criteria the location of the analyte in the time-mass-intensity plane (topographical map), the exact mass (ppm) and the deviation of the isotopic pattern in contrast to its theoretical value and quantified as mSigma by the T-Rex 3D algorithm (MetaboScape®). Thus, the values of 5 mDa and 50 mSigma are established as the maximum acceptable deviation of the mass of the compound and of the isotopic standard, respectively. In order to increase the reliability in the characterisation of the analytes, the scope of the calculation algorithm is expanded by integrating several search tools (Compound Crawler), fragmentation (MetFrag) and spectral comparison (MS/MS Bucket matches) that together provide a complete identification.

As a calculation example, the identification process of the analyte with m/z 463 detected exclusively in the enriched feed is shown. Initially, SmartFormula proposes the tentative molecular formula C_21_H_20_O_12_ , with the screening parameters mSigma < 50 and Δm/z < 5mDa. The identification is complemented by various spectral databases incorporated into MetaboScape®, proposing various linkages of the polyphenol quercetin to glycosides such as Quercetin-N-O-galactoside and Quercetin-N-O-glucoside. The glycosylated flavonoid potential shows the characteristic 300, 271, 255 and 253 m/z ions (**Section 1.1.A**). This mass profile was entered into the MoNA library obtaining a score >0.9 (unity being an exact similarity) for Quercetin-3-O-Galactoside with identifier MSBNK-RIKEN-PR100676.

After applying these external identification mechanisms, the proposed characterisation is validated using three different internal tools: MetFrag, MS/MS Bucket Matches and their location on the topographic map of analytes. MetFrag generates different proposals for *in silico* fragmentation of the molecule (**Section 1.1.B**). Thus, the Quercetin-3-O-Galactoside molecule responds to the characteristic ions of higher intensity, losing the galactose or glucose group (-162Da) for the 300 m/z ion, and the fragmentations of the 271 and 255 m/z ions originating from a typical ring opening in the quercetin fragmentation pathway.


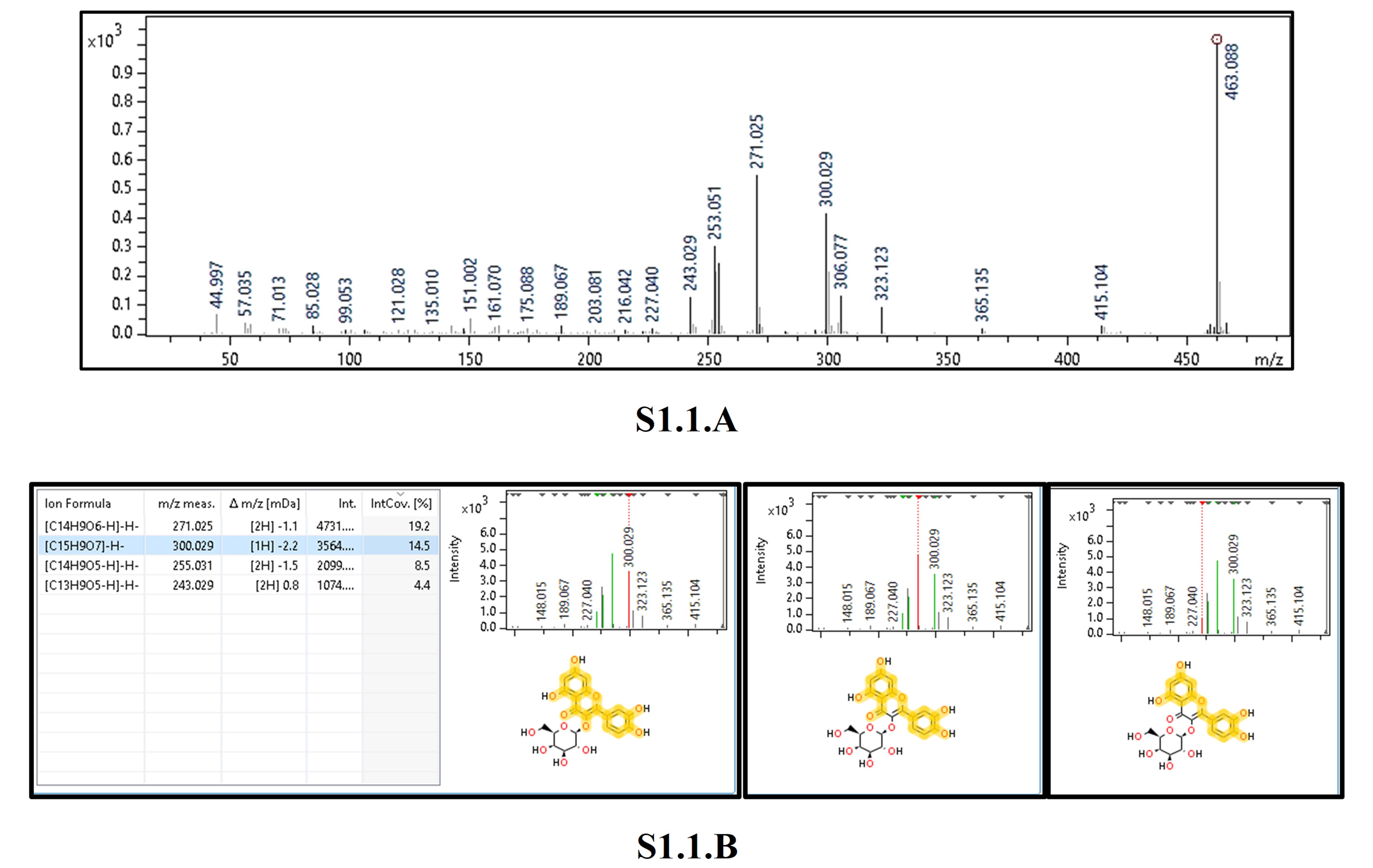


**1.1.A**


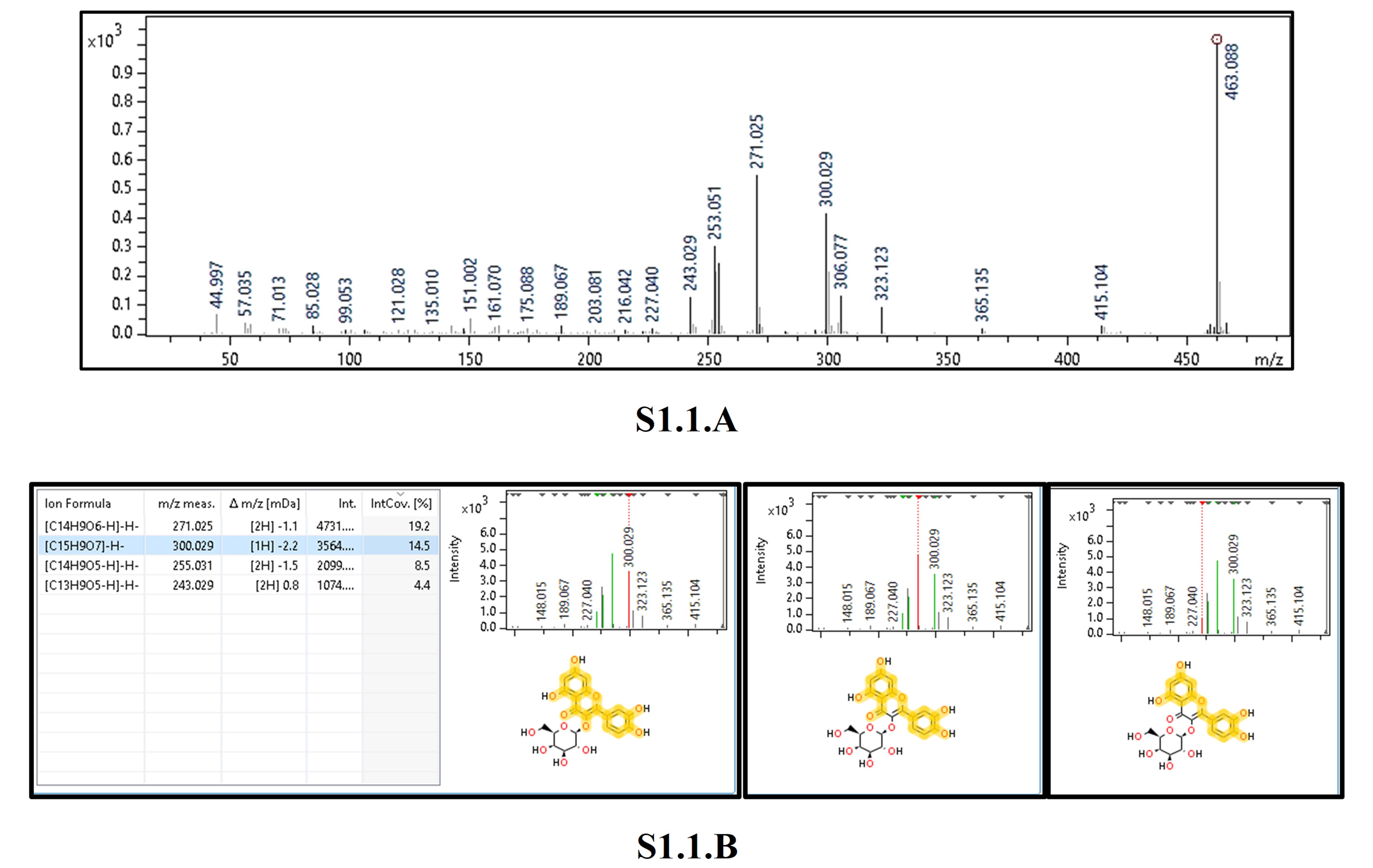


**1.1.B**

**Section 1.1. Untargeted identification of the compound Quercetin 3-O-Galactoside through its characteristic fragmentation. A) Mass spectrum of Quercetin 3-O-Galactoside, B) Fragment analysis by MetFrag.**

Moreover, all the analytes identified in the various samples are grouped together in a single container known as a "Bucket" in the MetaboScape® software. Therefore, when the mass profile of a compound is available, it is possible to compare it with the mass profiles of the different analytes in all the samples analysed. Using MS/MS Bucket Matches (**Section 1.2**), a clear correlation (r>0.9) is achieved between the analyte under study and glycosides of phenolic compounds previously identified as kaempferol-N-O-glucoside, gallic acid-N-O-glucoside and catechin-O-glucoside acetate, as well as polyphenolic structures such as epicatechin, and procyanidin B1 dimers.


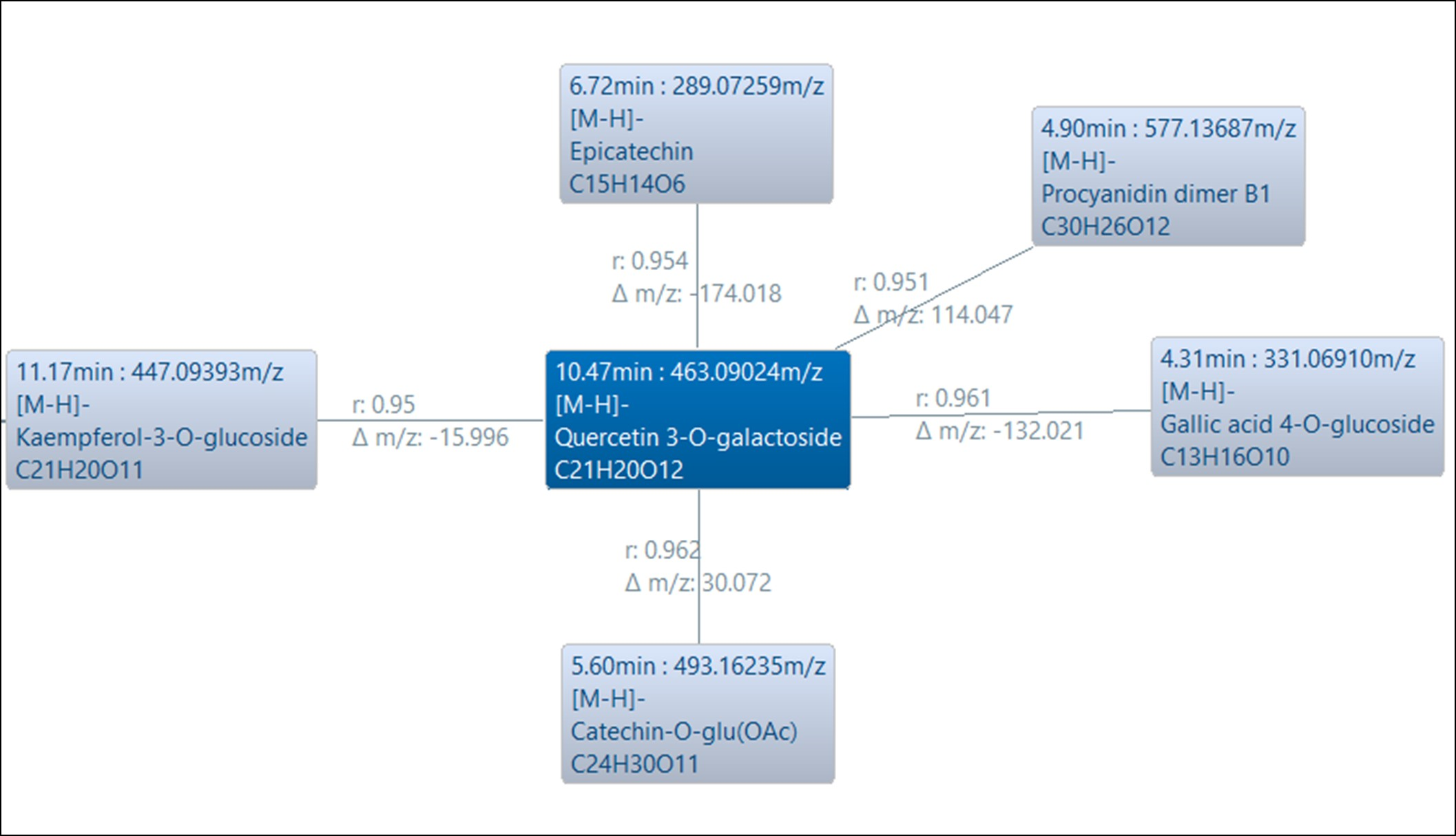


**Section 1.2. Spectral correlation through MS/MS bucket matches of the 463 m/z ion, tentatively identified as Quercetin 3-O-Galactoside.**

Regarding the topographical map (**Section 1.3**), a similar structure to the results of the MS/MS Bucket match analysis is observed, evidencing parallelisms in compound intensity (represented by the size of the dots), retention times and mass-to-charge ratios (m/z); of other glycosylated phenolics. In this sense, the topographical map highlights a specific zone containing compounds such as apigenin glycosides, quercetin, naringenin and kaempferol, which reinforces the proposed identification of a phenolic structure linked to a glycosylated derivative.

In this context, the conjunction of the identification tools shows the ability of this methodology to narrow down the analytes to a base structure, making it an exceptionally robust analysis. However, the list of potential isomeric candidates for naming structures is generally extensive, especially when assessing the different forms in which polyphenolic chains are presented, as well as potential variations in glycosylation. This diversity contributes to the complexity of achieving a complete and unambiguous identification, thus establishing compounds characterised with the designation "isomer" when their structure fits more than one molecular distribution and as "derivative" when identification methods can only classify the analyte as part of a family of compounds or derivative of a particular compound.


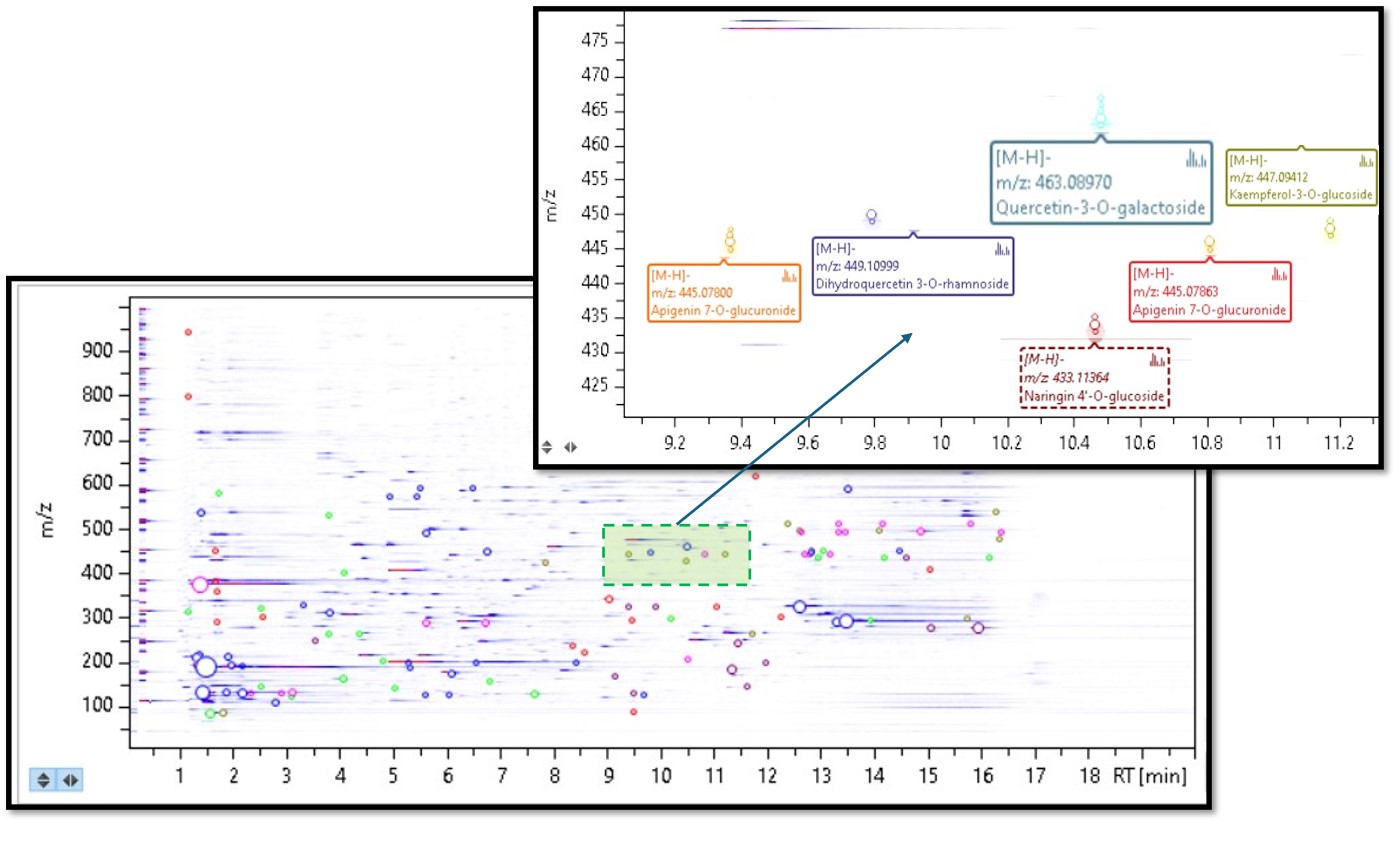


**Section 1.3. Model topographic map of the e-Vitis feed, with emphasis on the glycosylated polyphenol region**.

**Section 2. Statistical study parameters via Metaboanalyst 6.0**

All data were filtered using the relative standard deviation RSD>25% and normalised by summation. The separation of the data corresponding to the base and e-Vitis-enriched feeds, as well as to the base ingredients, was carried out using Partial Least Squares - Discriminant Analysis (PLS-DA). For the selection of representative compounds in each group, a one-way ANOVA with false discovery rate (FDR) p<0.05 was performed. For the biological samples, a targeted Orthogonal Partial Least Squares - Discriminant Analysis (OPLS-DA) was established. Data segmentation in this case was by Fold Change (FC) Analysis, with FC equal to 2 and with mean ratio of each group M1/M2>2 or M1/M2<1/2. By means of the 5-component evaluation, the PC1 and PC2 values were established which gave the best discrimination and representation of the data. The predictive capacity of the model Q2 and the determination coefficient R2Y were determined as model evaluation parameters.

**Section 3. Statistical and validation parameters applied**

The metabolic segmentation and pooling quality of the different study groups, for the case of feed and feed ingredients for swine (Ctrl Feed, e-Vitis Feed, Beetroot and Soybean Meal groups) was quantified by a directed PLS-DA analysis, represented in the score plot in Fig. S3.1, obtaining fit parameters R2Y > 0.998, Q2>0.998, and p < 5E-04 (0/2000); as well as an ANOVA analysis for the identification of representative compounds (p<0.05). In the case of the Standard Feed and e-Vitis Feed groups, a supervised OPLS-DA analysis was performed Fig. S3.2-9 obtaining global fit parameters R2Y > 0.968, Q2>0.989, and p < 5E-04 (0/2000). Similarly, orthogonal score plots are plotted as the t-analysis represented in the volcano plot highlighting the characteristic points (markers) of each group (p<0.05).

**Section 4. Statistical and validation parameters applied to samples of standard feed ingredients, and feed (4.1), and digestive tract and excreta: gastric (4.2), duodenum (4.3), ileum (4.4), jejunum (4.5), caecum (4.6), colon (4.7), faeces (4.8) and urine (4.9) of pigs.**

| **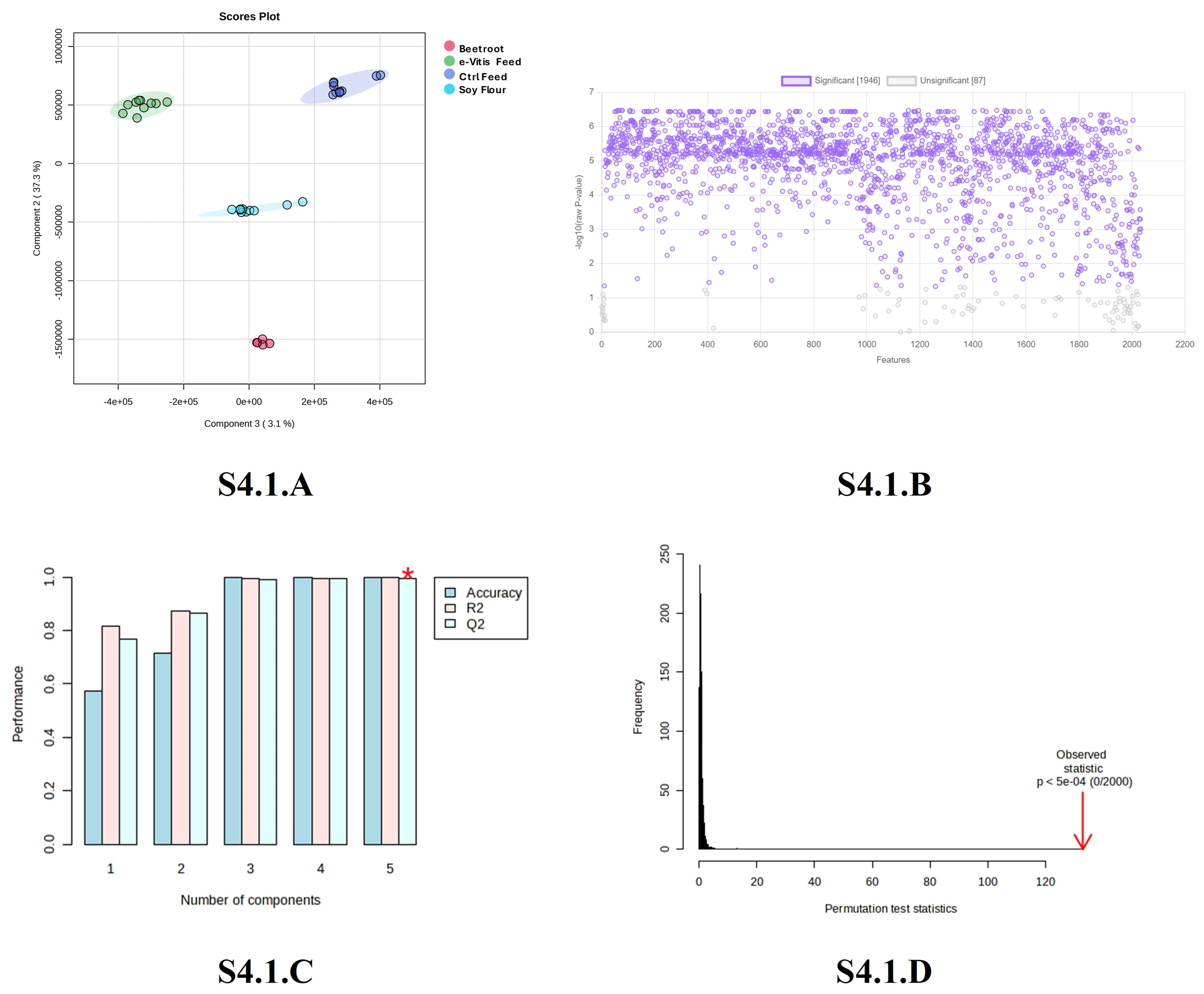** | |
| --- | --- |
| **4.1.A** | **4.1.B** |
| **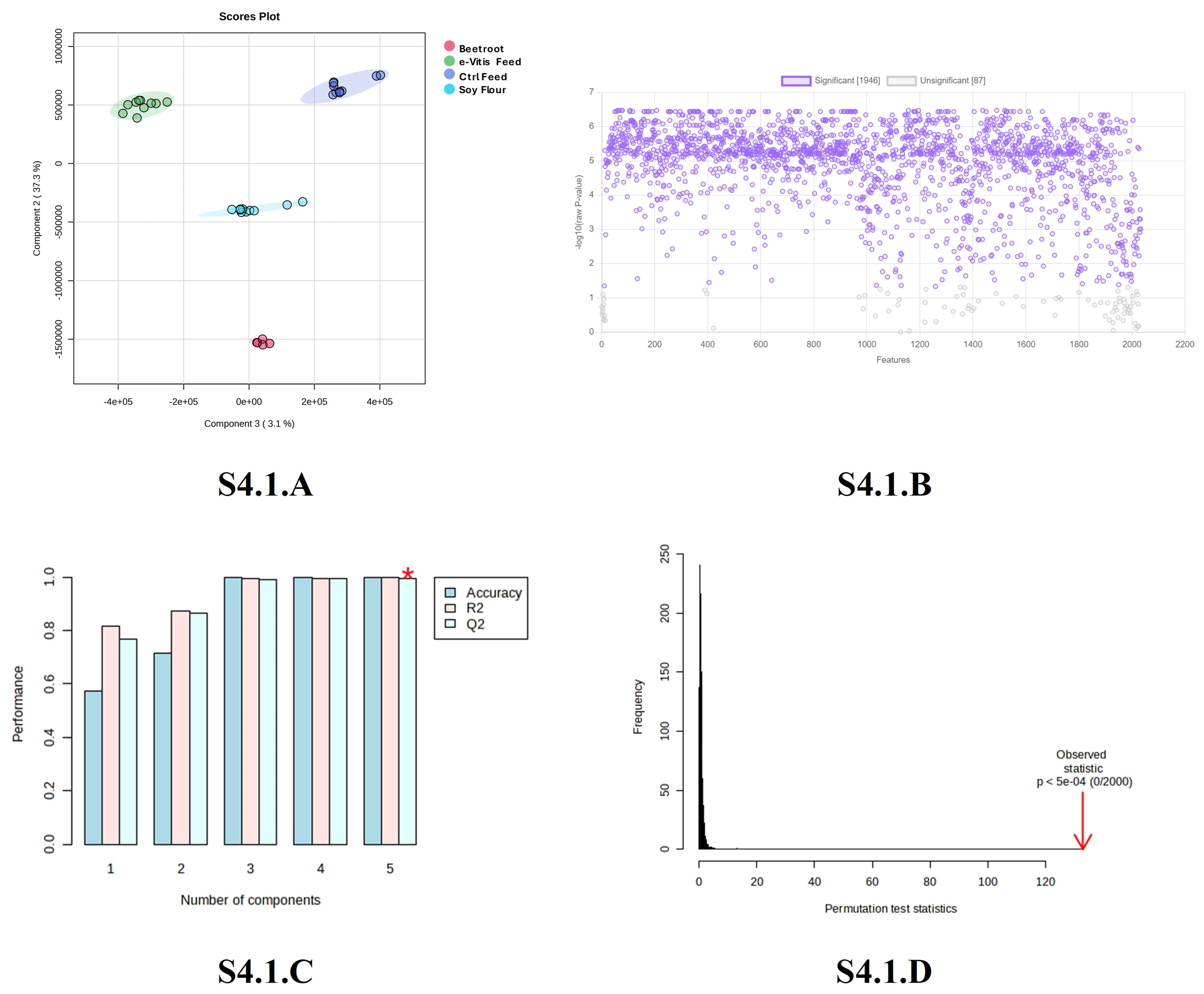** | |
| **4.1.C** | **4.1.D** |

**Section 4.1. Metabolomics analysis of standard feed ingredients, and feed.** Partial least squares discriminant analysis (PLS-DA) (**4.1.A**), one-way ANOVA (**4.1.B**), optimal number of components for PLS-DA classification by cross-validation (**4.1.C**) and permutation of 2000 iterations (**4.1.D**). Ctrl Feed: base feed; e-Vitis feed: feed enriched with e-Vitis.

| 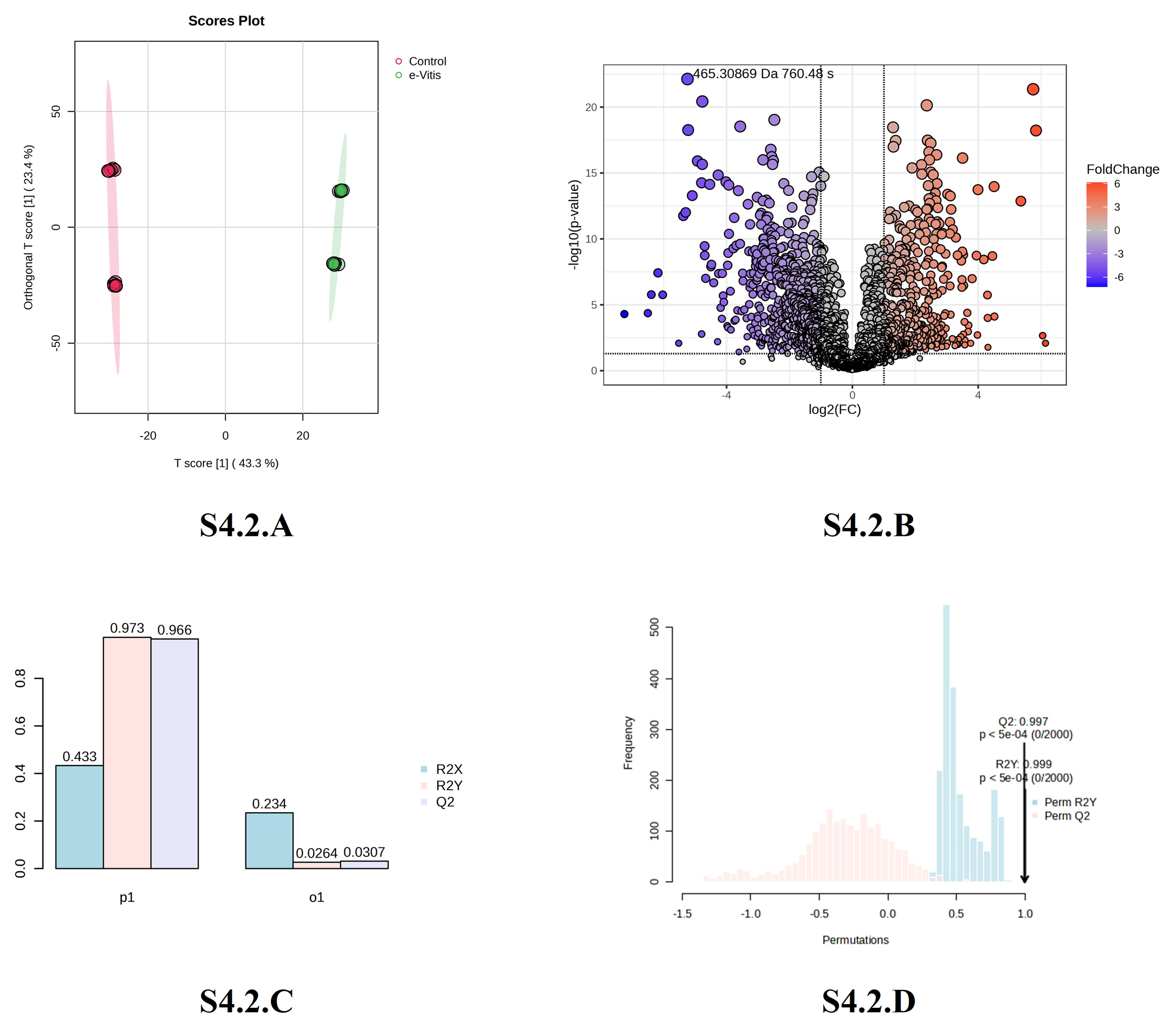 | |
| --- | --- |
| **4.2.A** | **4.2.B** |
| **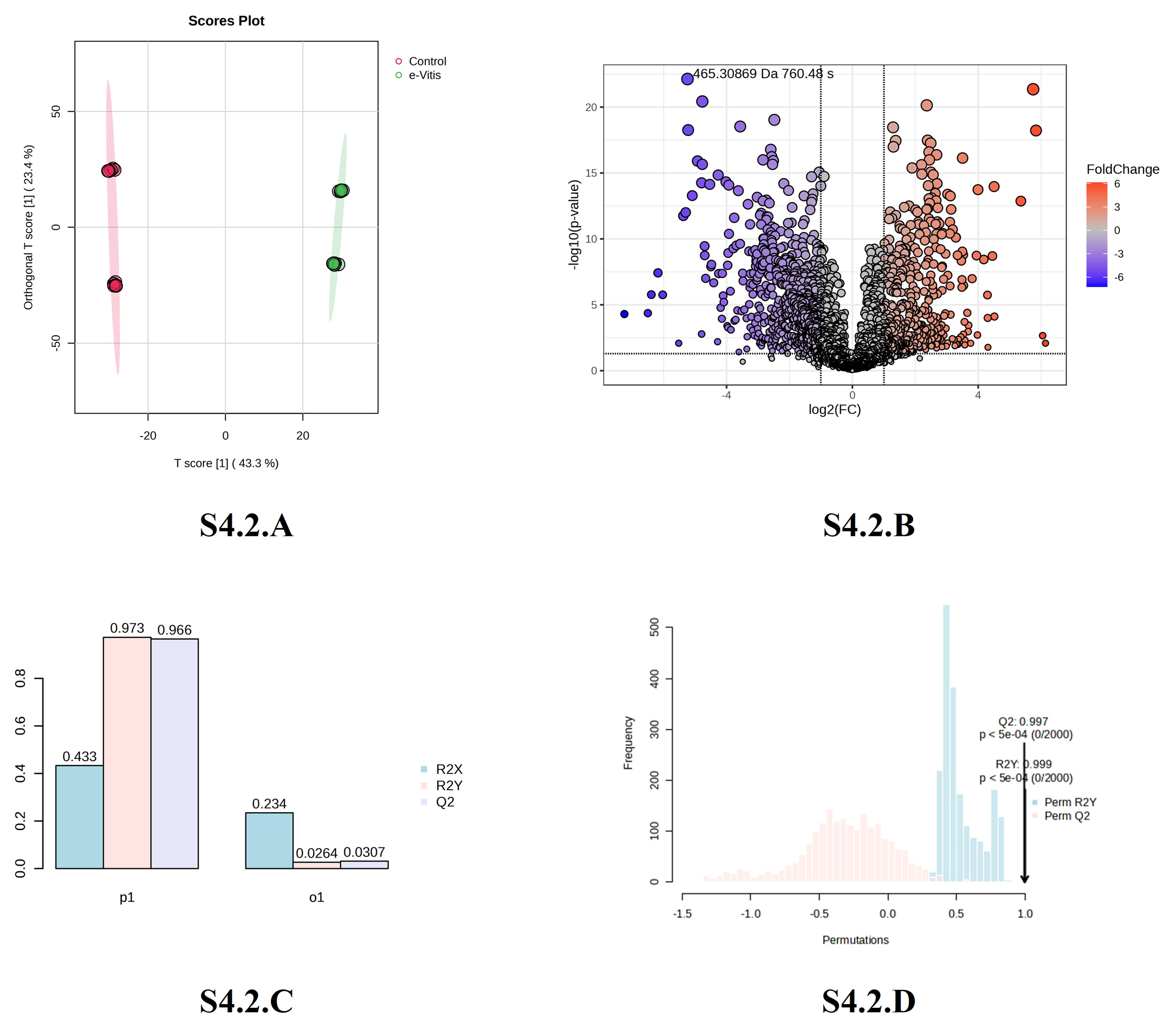** | |
| **4.2.C** | **4.2.D** |

**Section 4.2. Metabolomics analysis of Gastric Content.** Orthogonal partial least squares discriminant analysis (OPLS-DA) (**4.2.A**), volcano plot (**4.2.B**), optimal number of components for OPLS-DA classification by cross-validation (**4.2.C**) and permutation of 2000 iterations (**4.2.D**). e-Vitis: pigs fed with enriched feed. Ctrl: pigs fed with base feed.

| 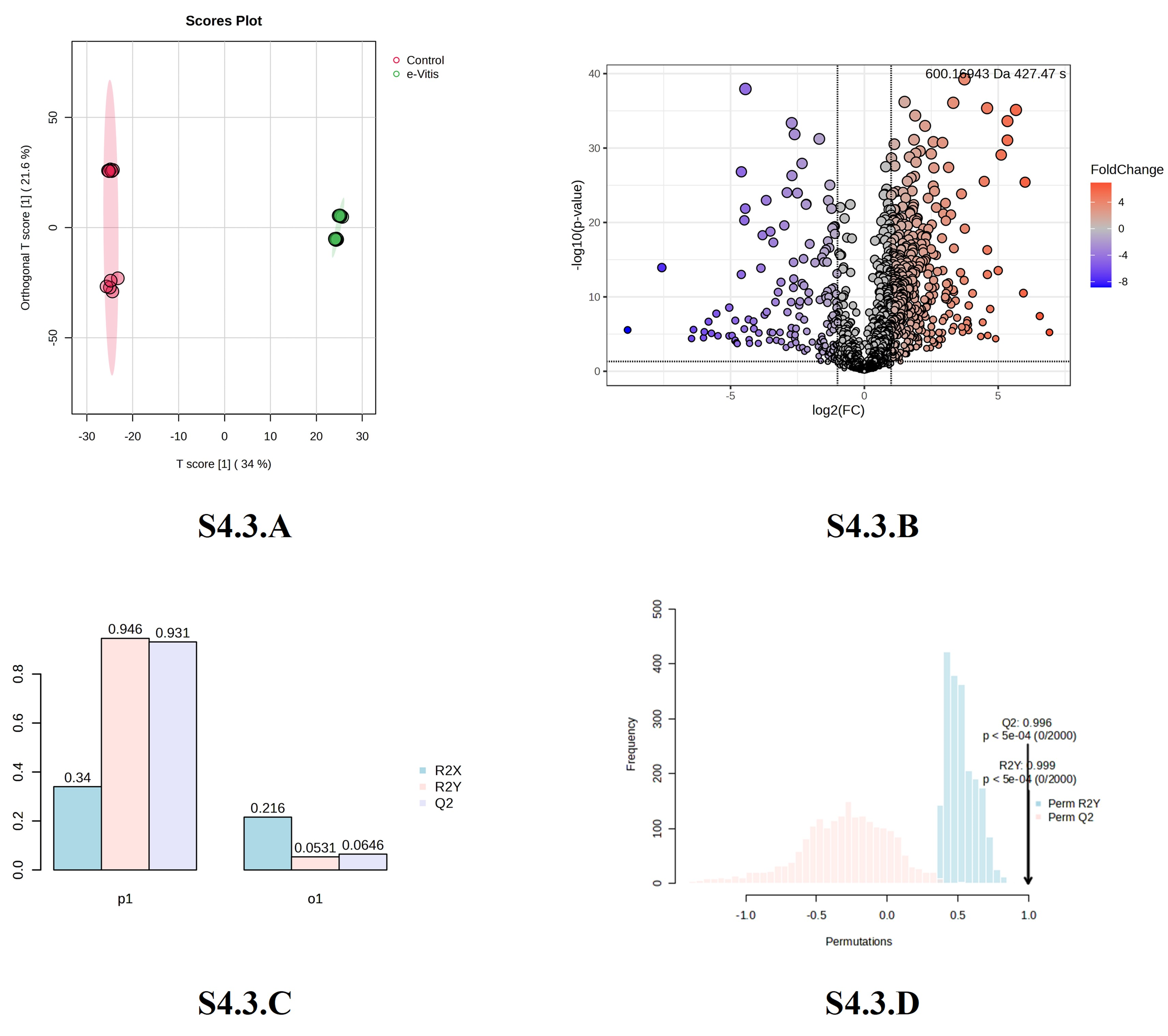 | |
| --- | --- |
| **4.3.A** | **4.3.B** |
| 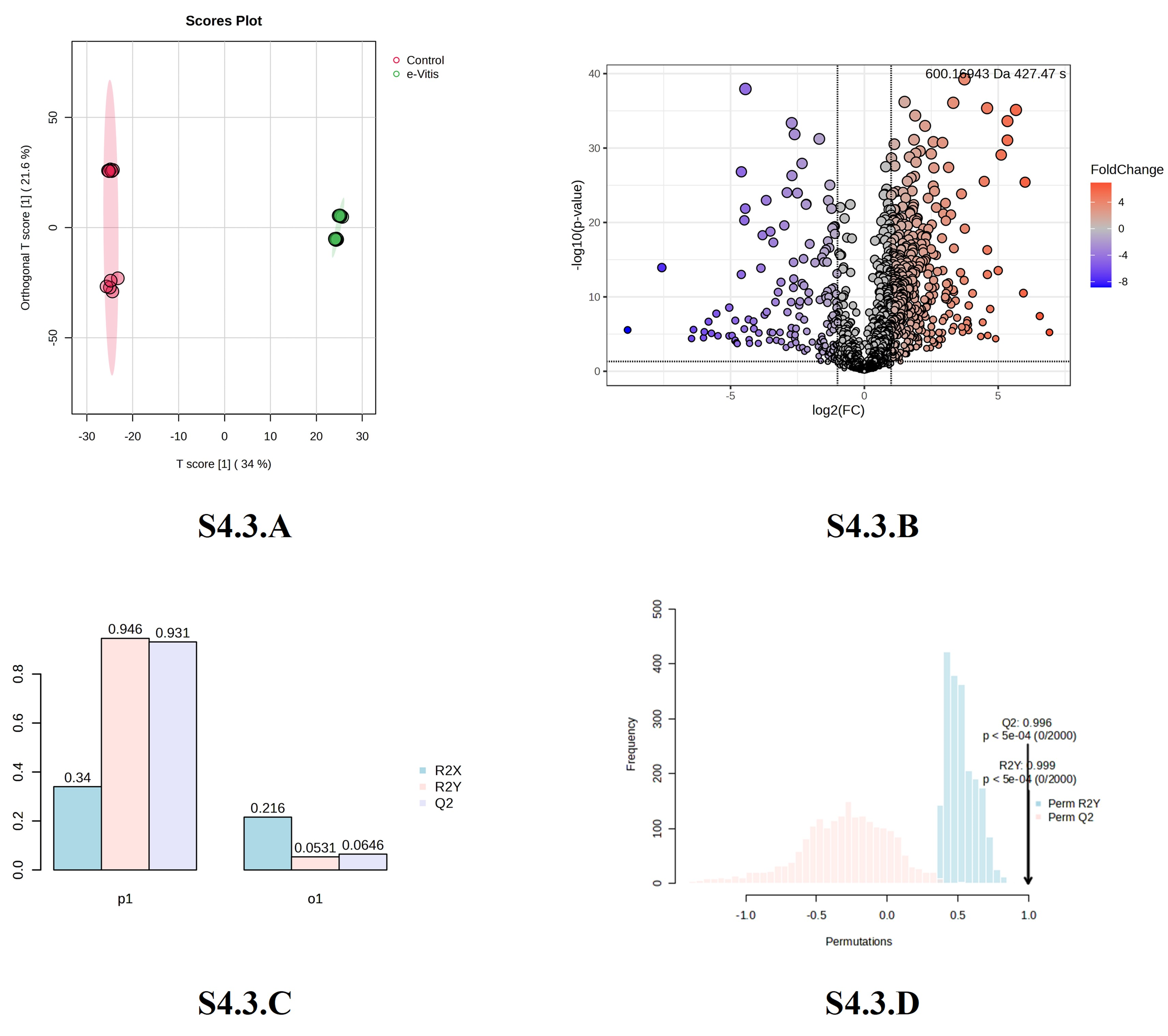 | |
| **4.3.C** | **4.3.D** |

**Section 4.3. Metabolomics analysis of duodenum.** Orthogonal partial least squares discriminant analysis (OPLS-DA) (**4.3.A**), volcano plot (**4.3.B**), optimal number of components for OPLS-DA classification by cross-validation (**4.3.C**) and permutation of 2000 iterations (**4.3.D**). e-Vitis: pigs fed with enriched feed. Ctrl: pigs fed with base feed.

| 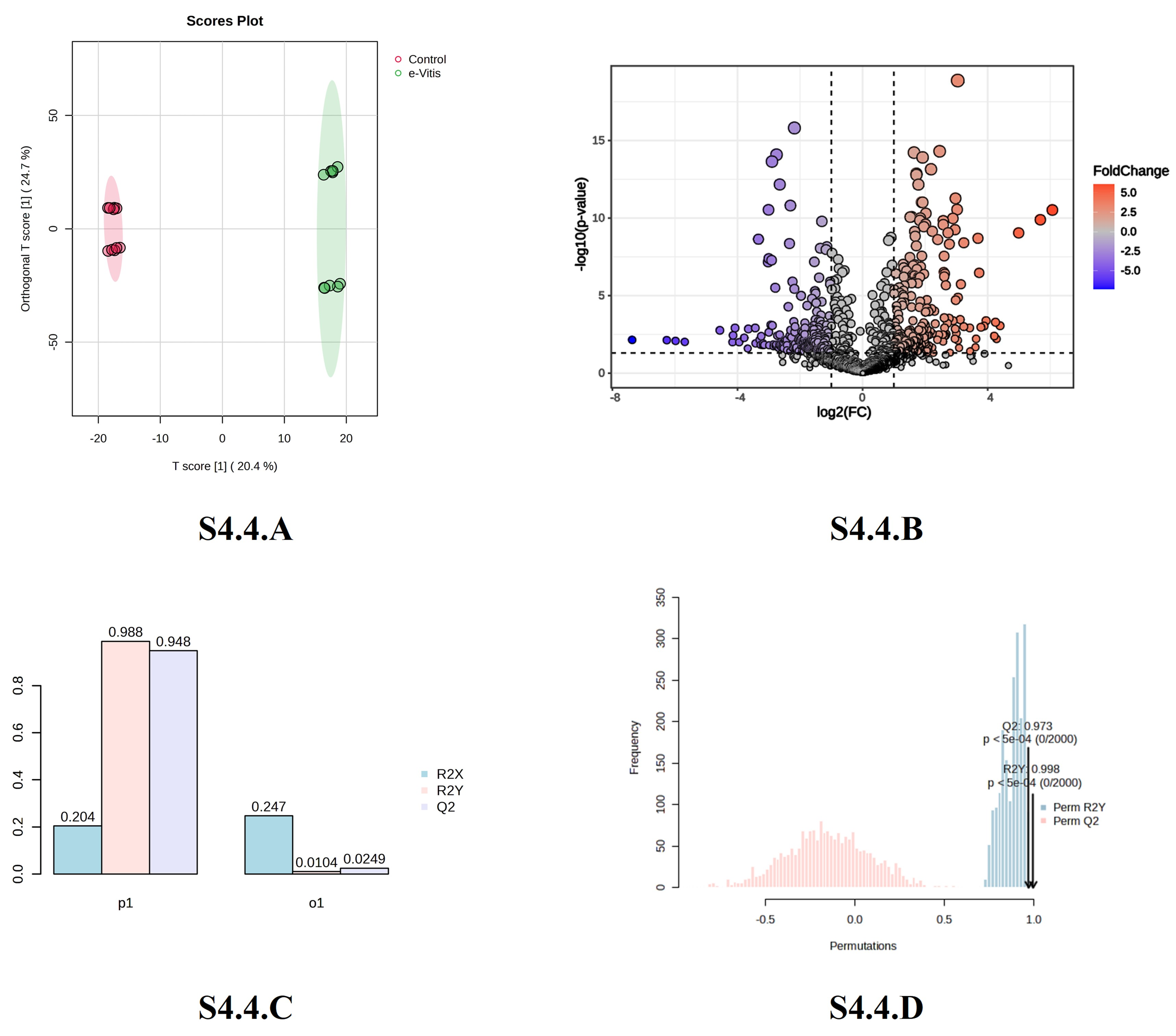 | |
| --- | --- |
| **4.4.A** | **4.4.B** |
| 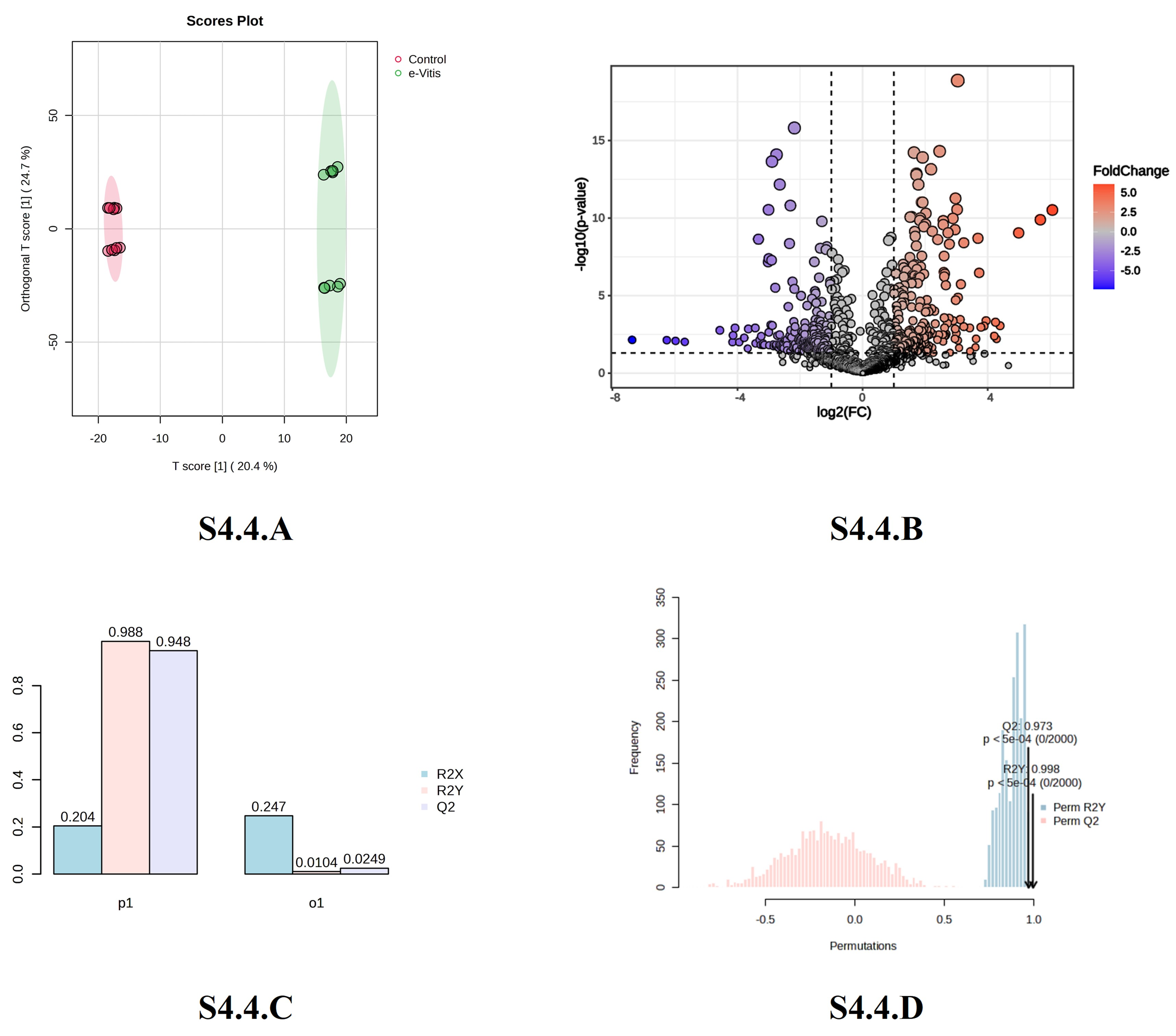 | |
| **4.4.C** | **4.4.D** |

**Section 4.4. Metabolomics analysis of ileum.** Orthogonal partial least squares discriminant analysis (OPLS-DA) (**4.4.A**), volcano plot (**4.4.B**), optimal number of components for OPLS-DA classification by cross-validation (**4.4.C**) and permutation of 2000 iterations (**4.4.D**). e-Vitis: pigs fed with enriched feed. Ctrl: pigs fed with base feed.

| 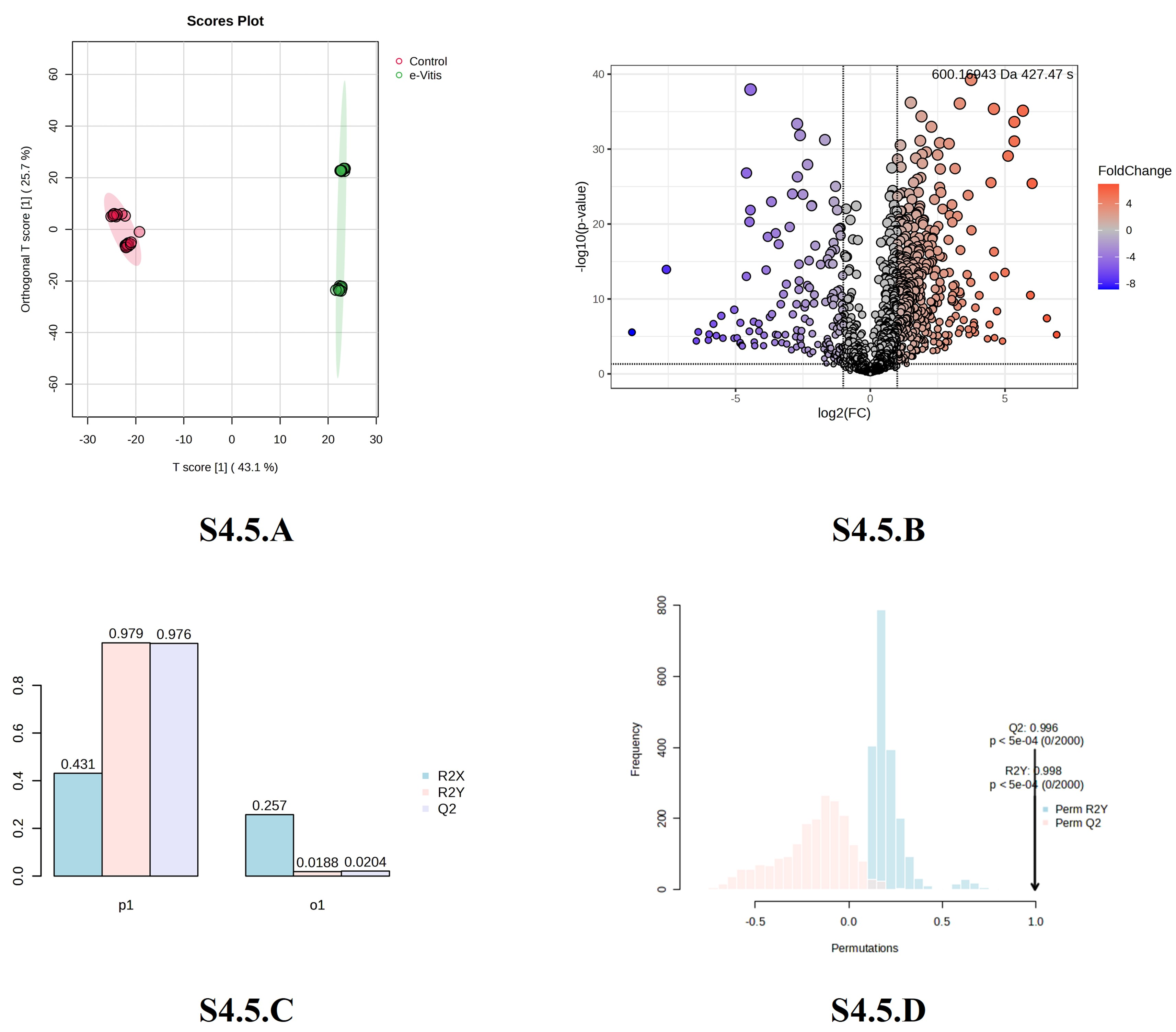 | |
| --- | --- |
| **4.5.A** | **4.5.B** |
| 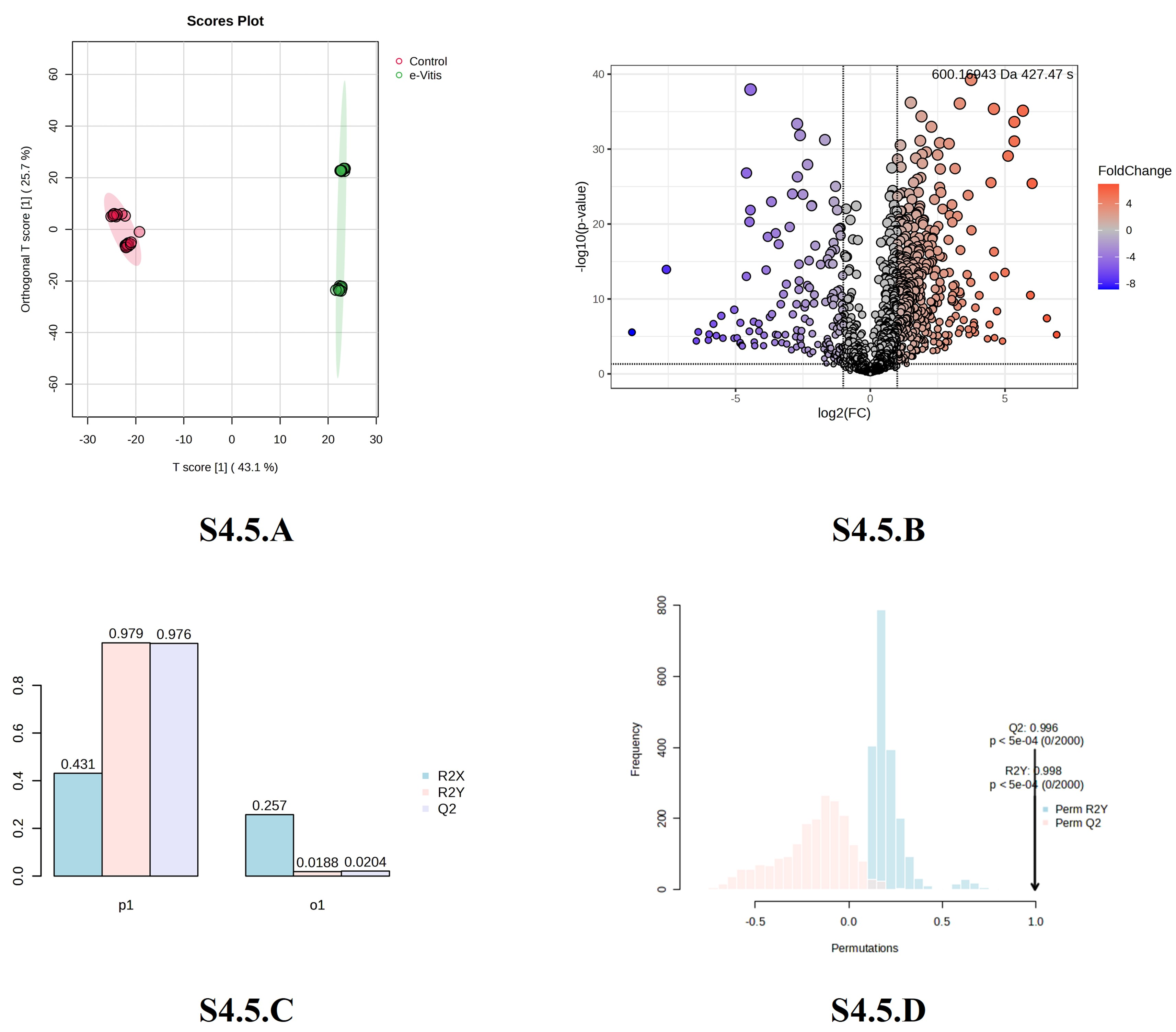 | |
| **4.5.C** | **4.5.D** |

**Section 4.5. Metabolomics analysis of jejunum.** Orthogonal partial least squares discriminant analysis (OPLS-DA) (**4.5.A**), volcano plot (**4.5.B**), optimal number of components for OPLS-DA classification by cross-validation (**4.5.C**) and permutation of 2000 iterations (**4.5.D**). e-Vitis: pigs fed with enriched feed. Ctrl: pigs fed with base feed.

| 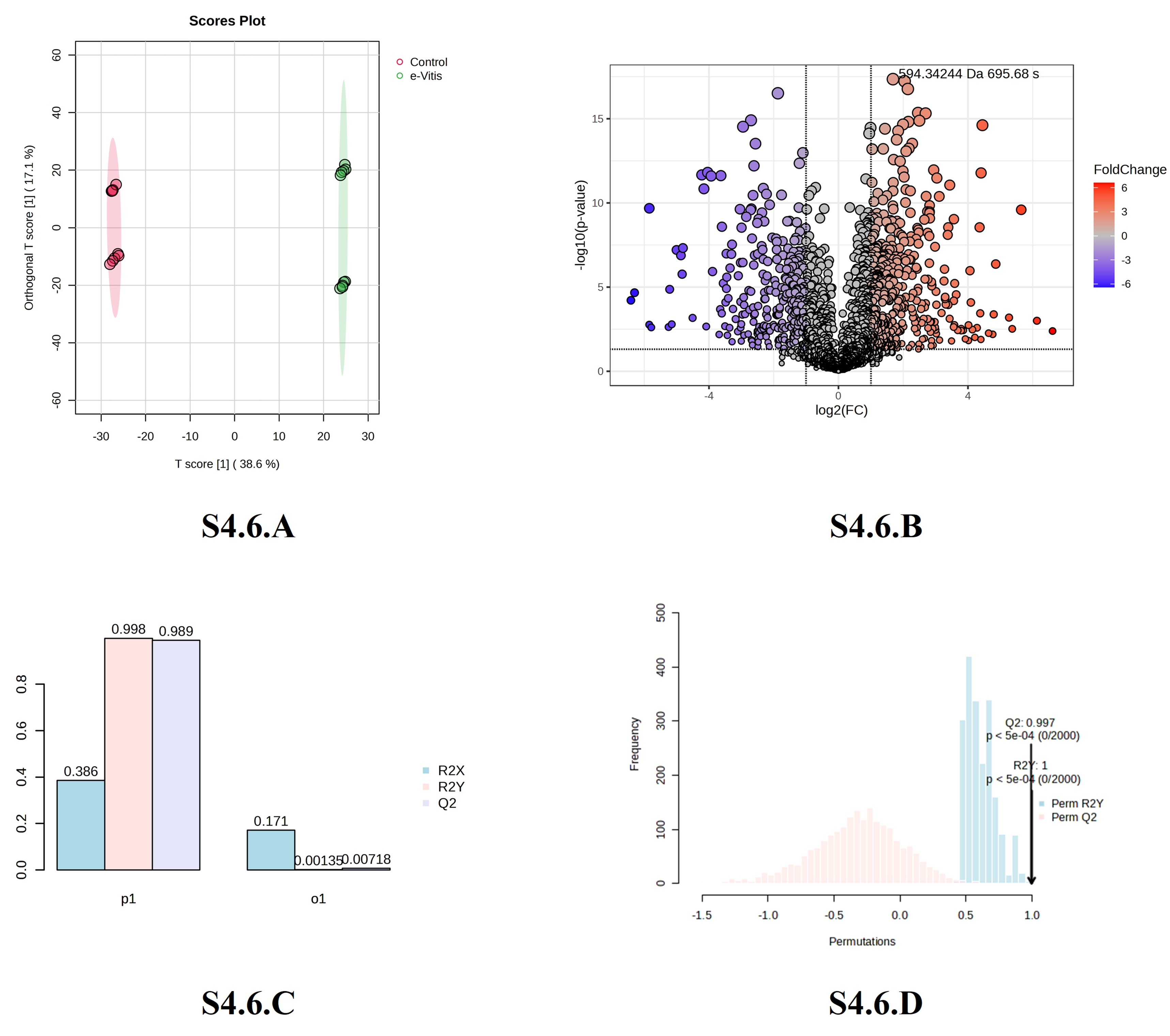 | |
| --- | --- |
| **4.6.A** | **4.6.B** |
| 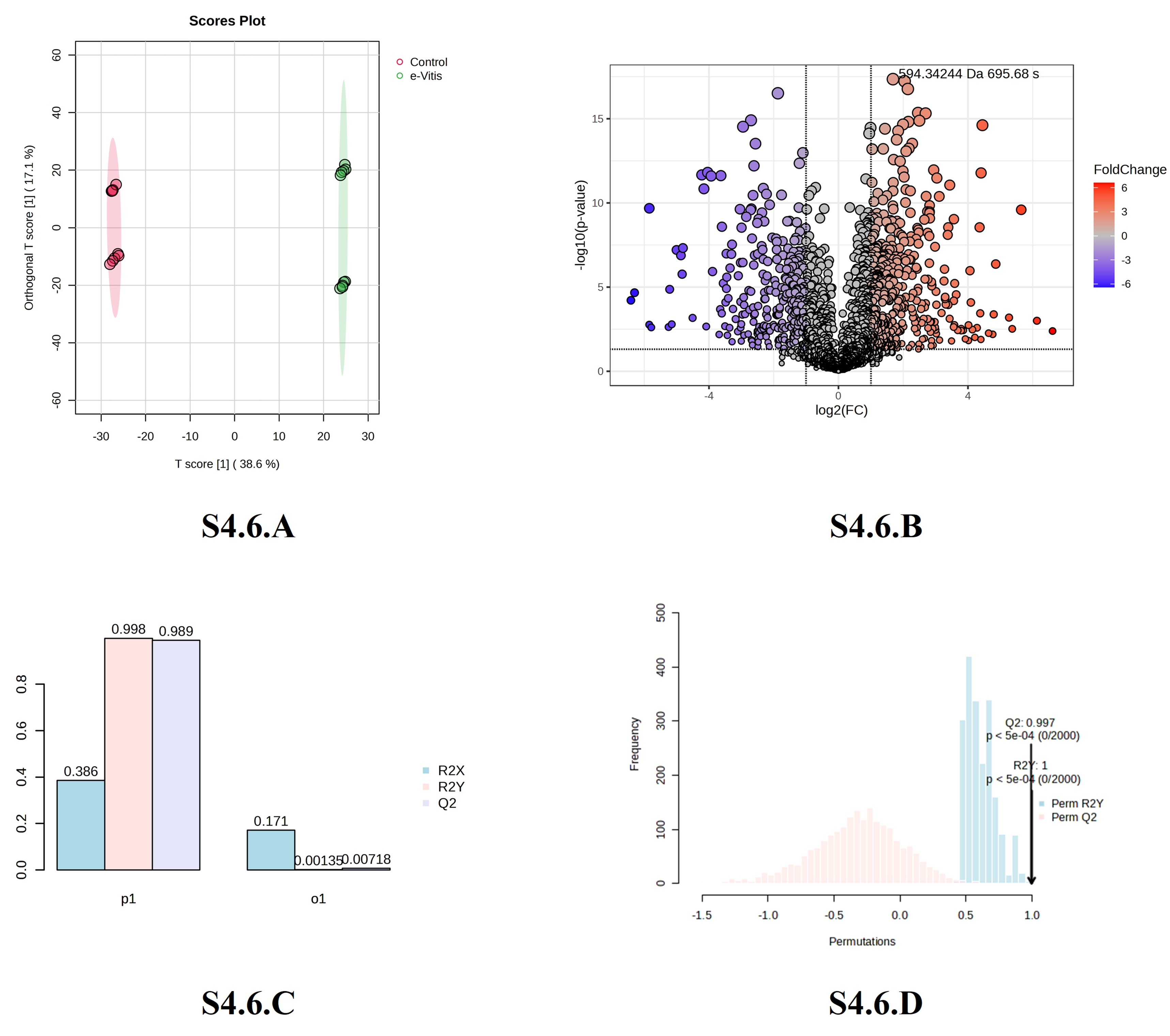 | |
| **4.6.C** | **4.6.D** |

**Section 4.6. Metabolomics analysis of caecum.** Orthogonal partial least squares discriminant analysis (OPLS-DA) (**4.6.A**), volcano plot (**4.6.B**), optimal number of components for OPLS-DA classification by cross-validation (**4.6.C**) and permutation of 2000 iterations (**4.6.D**). e-Vitis: pigs fed with enriched feed. Ctrl: pigs fed with base feed.

| 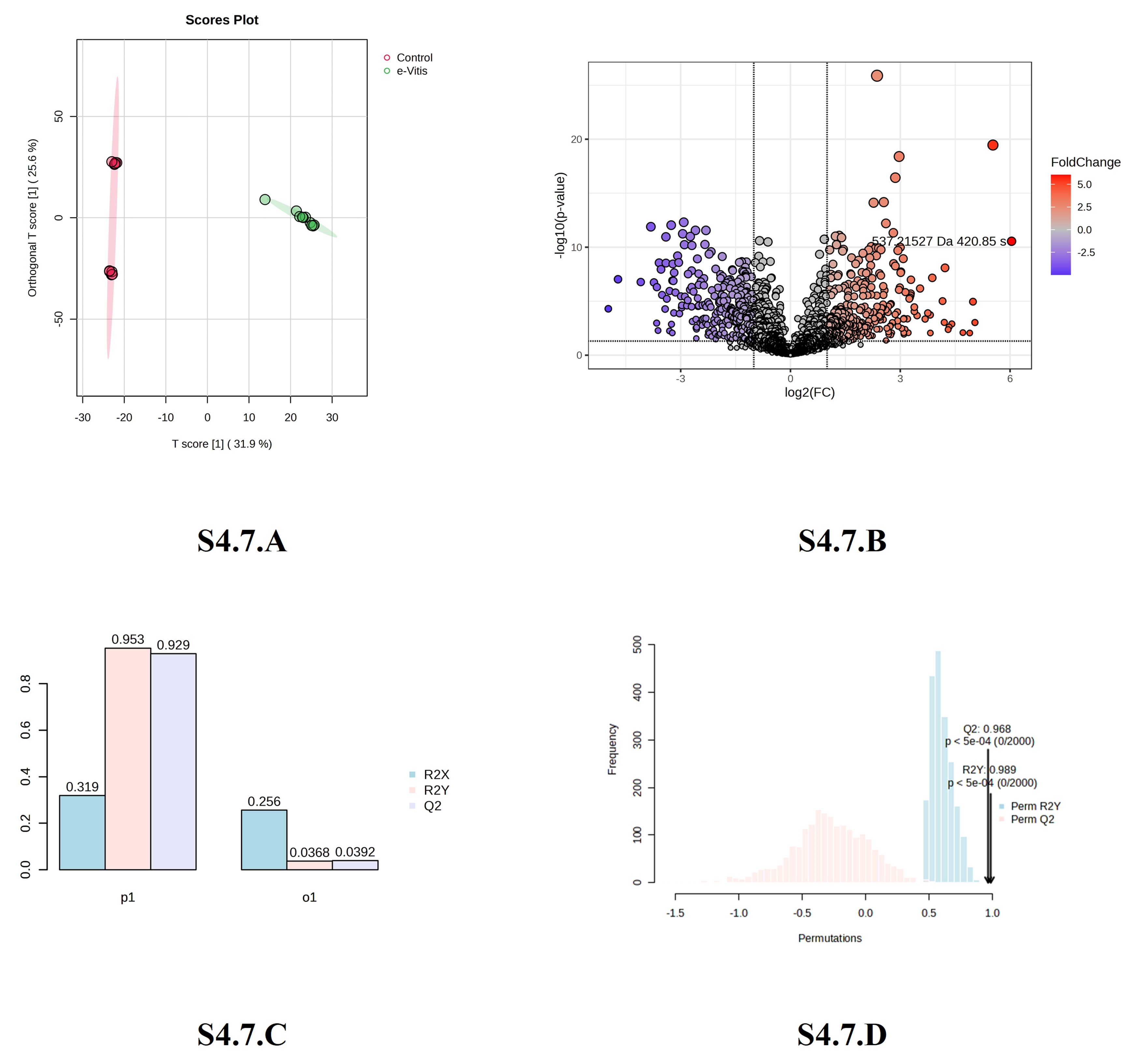 | |
| --- | --- |
| **4.7.A** | **4.7.B** |
| 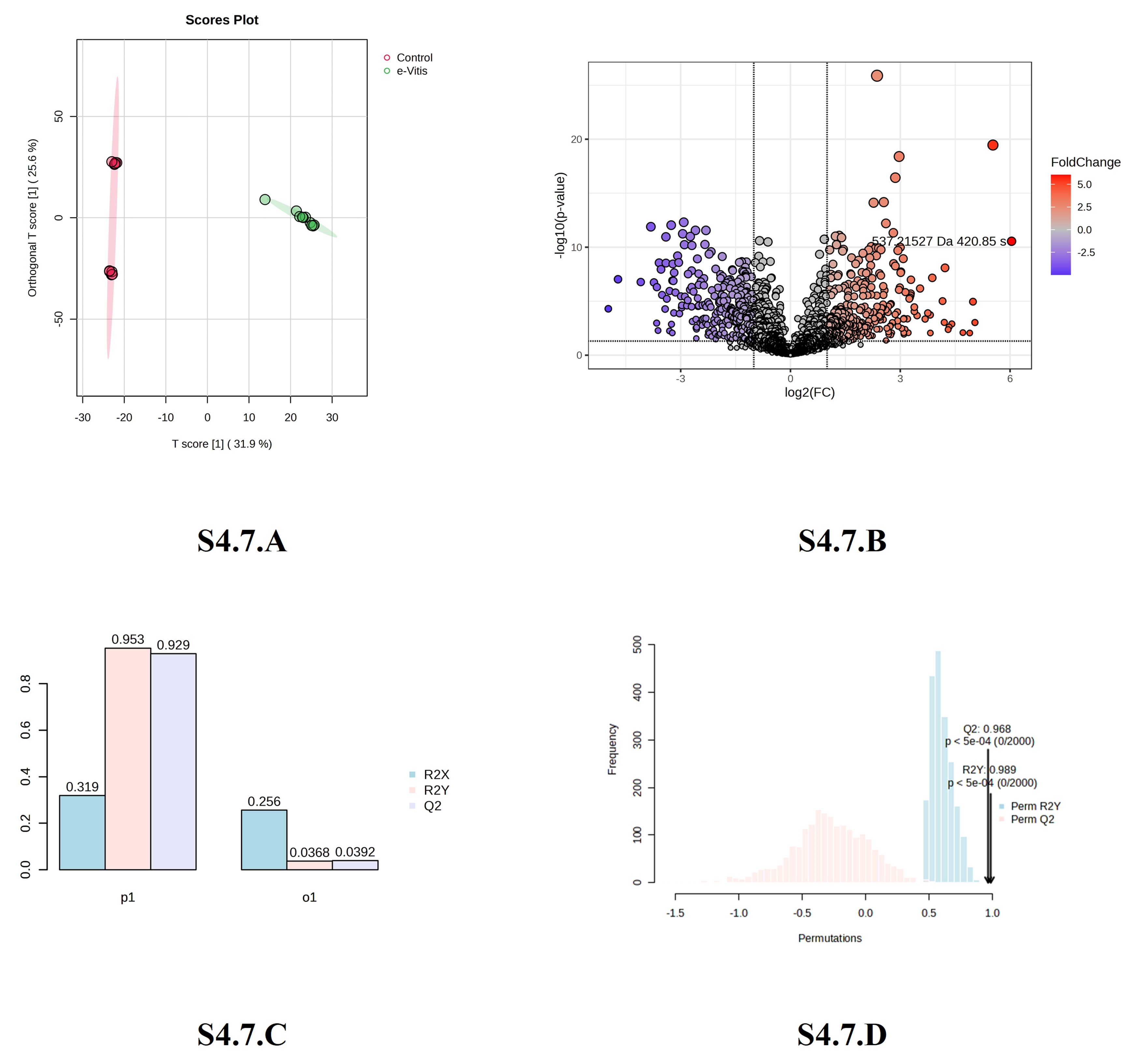 | |
| **4.7.C** | **4.7.D** |

**Section 4.7. Metabolomics analysis of colon.** Orthogonal partial least squares discriminant analysis (OPLS-DA) (**4.7.A**), volcano plot (**4.7.B**), optimal number of components for OPLS-DA classification by cross-validation (**4.7.C**) and permutation of 2000 iterations (**4.7.D**). e-Vitis: pigs fed with enriched feed. Ctrl: pigs fed with base feed.

| 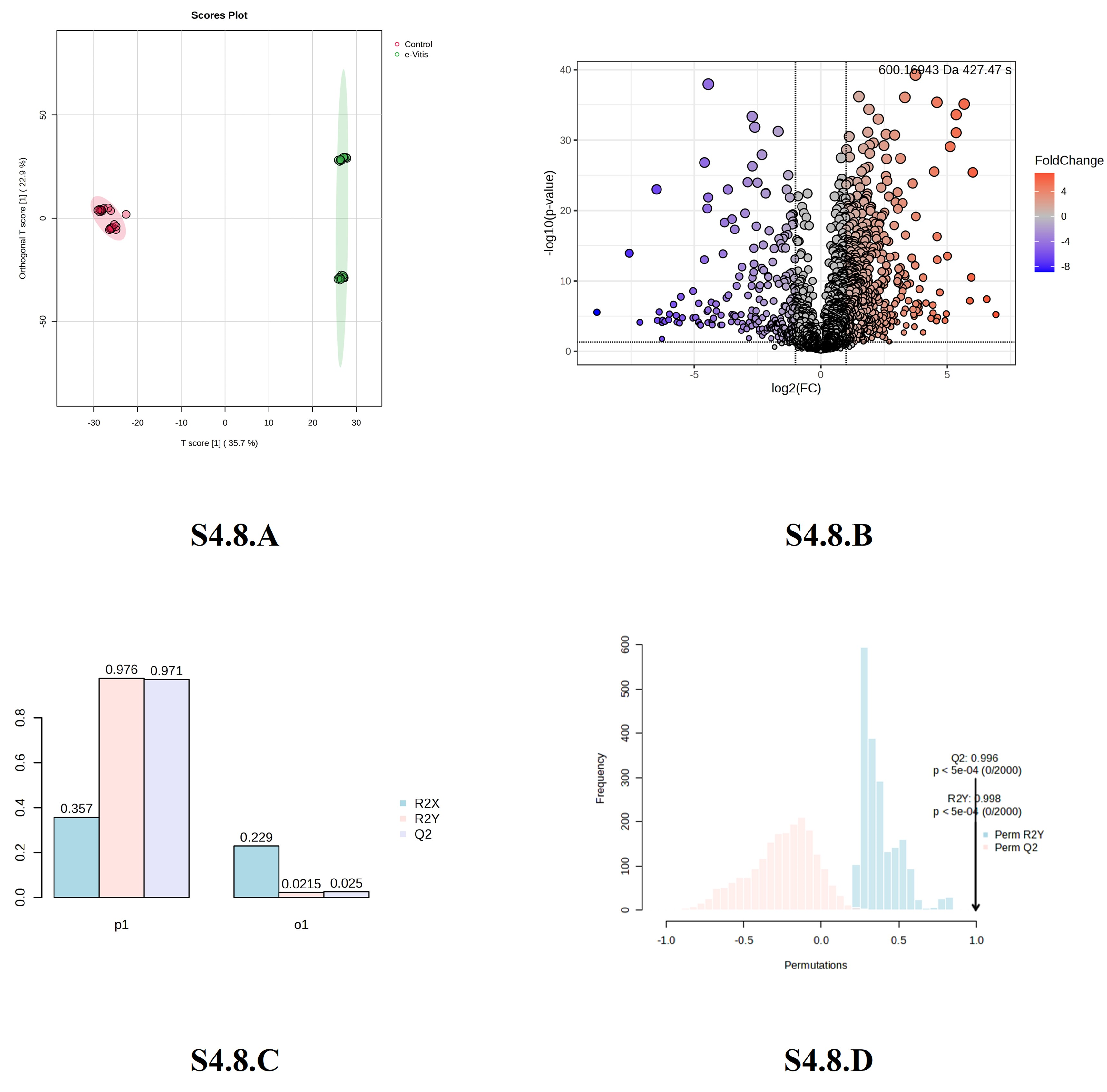 | |
| --- | --- |
| **4.8.A** | **4.8.B** |
| 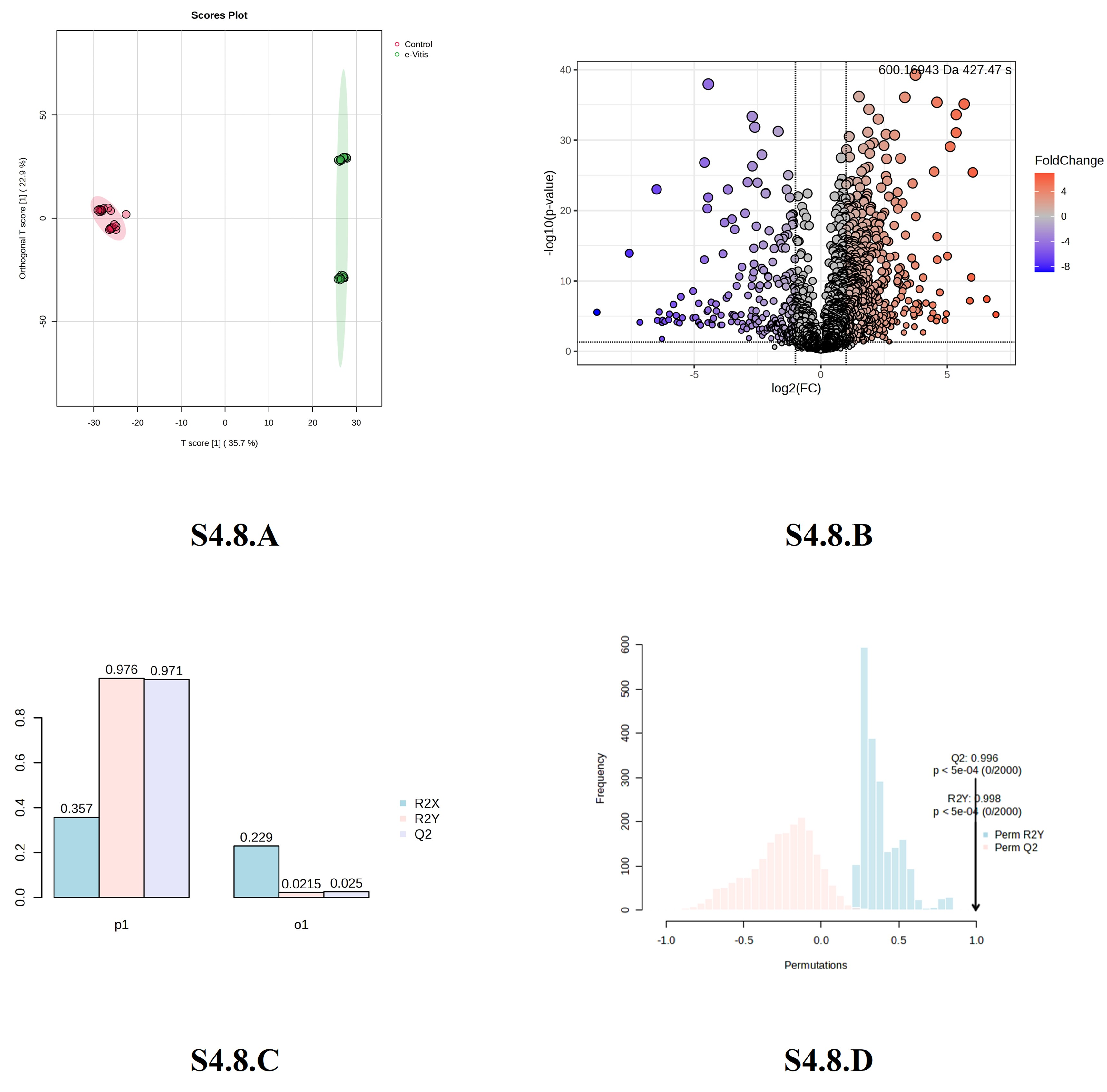 | |
| **4.8.C** | **4.8.D** |

**Section 4.8. Metabolomics analysis of urine.** Orthogonal partial least squares discriminant analysis (OPLS-DA) (**4.8.A**), volcano plot (**4.8.B**), optimal number of components for OPLS-DA classification by cross-validation (**4.8.C**) and permutation of 2000 iterations (**4.8.D**). e-Vitis: pigs fed with enriched feed. Ctrl: pigs fed with base feed.

| 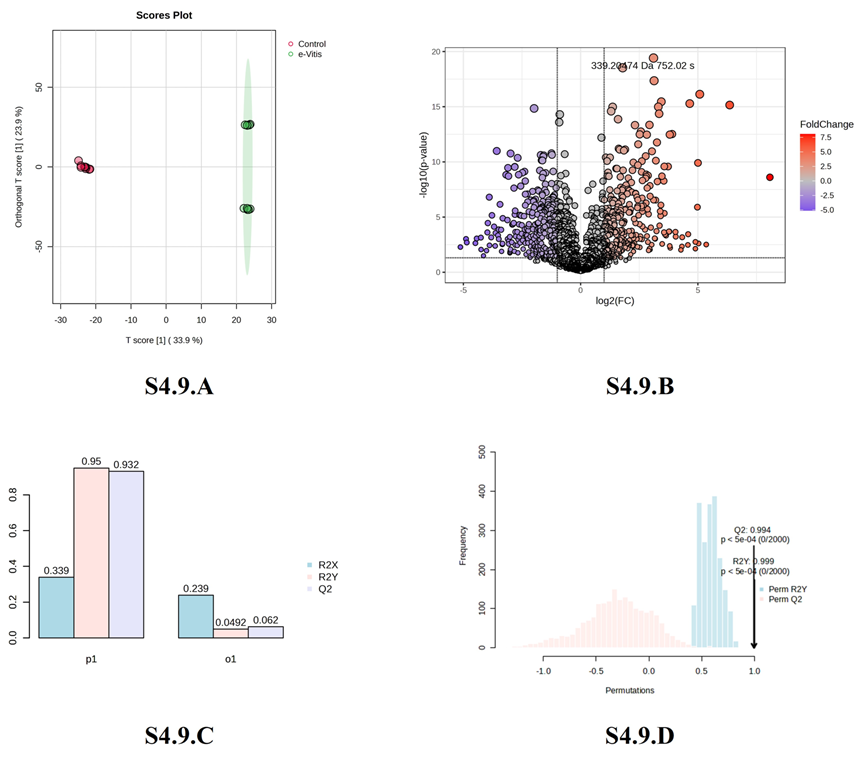 | |
| --- | --- |
| **4.9.A** | **4.9.B** |
| 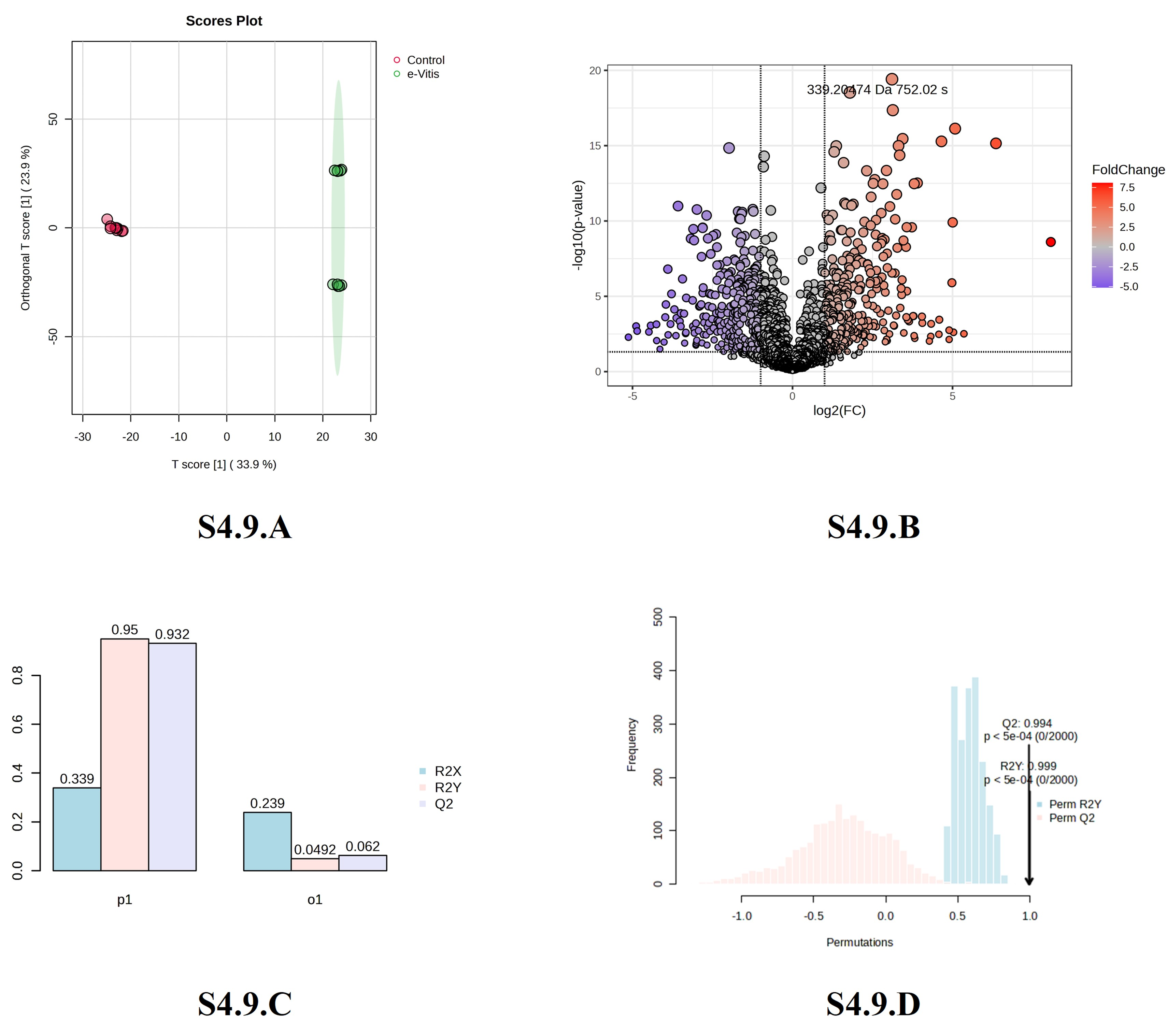 | |
| **4.9.C** | **4.9.D** |

**Section 4.9. Metabolomics analysis of faeces.** Orthogonal partial least squares discriminant analysis (OPLS-DA) (**4.9.A**), volcano plot (**4.9.B**), optimal number of components for OPLS-DA classification by cross-validation (**4.9.C**) and permutation of 2000 iterations (**4.9.D**). e-Vitis: pigs fed with enriched feed. Ctrl: pigs fed with base feed.
